# The Role of Surface Chemistry in the Orientational Behavior of Water at an Interface

**DOI:** 10.1021/acs.jpcb.2c01752

**Published:** 2022-06-21

**Authors:** Rowan Walker-Gibbons, Alžbeta Kubincová, Philippe H. Hünenberger, Madhavi Krishnan

**Affiliations:** † Physical & Theoretical Chemistry Laboratory, Department of Chemistry, University of Oxford, South Parks Road, Oxford OX1 3QZ, United Kingdom; ‡ Laboratory of Physical Chemistry, Department of Chemistry and Applied Biosciences, ETH Zurich, Vladimir-Prelog-Weg 2, CH-8093 Zürich, Switzerland

## Abstract

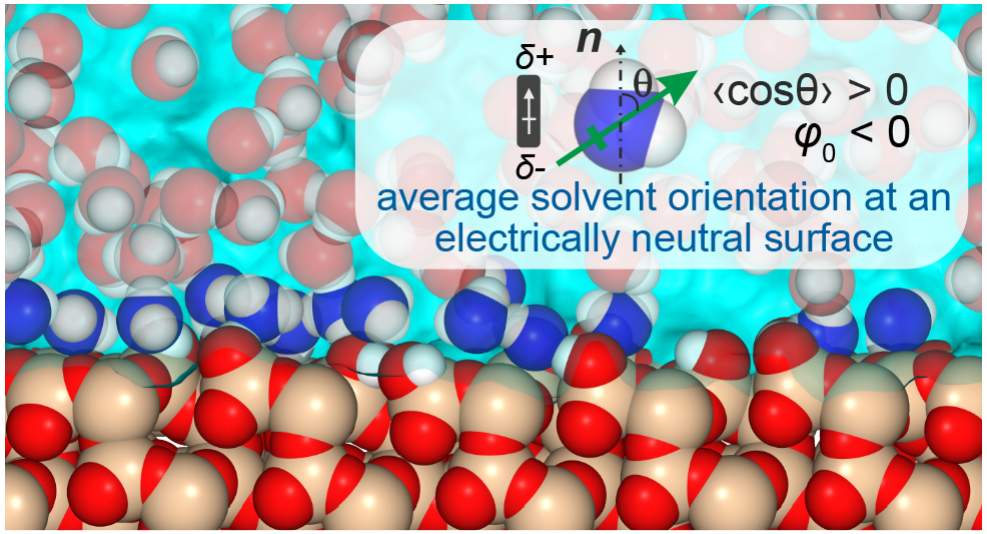

Molecular dynamics studies have demonstrated that molecular water at an interface, with either a gas or a solid, displays anisotropic orientational behavior in contrast to its bulk counterpart. This effect has been recently implicated in the like-charge attraction problem for colloidal particles in solution. Here, negatively charged particles in solution display a long-ranged attraction where continuum electrostatic theory predicts monotonically repulsive interactions, particularly in solutions with monovalent salt ions at low ionic strength. Anisotropic orientational behavior of solvent molecules at an interface gives rise to an excess interfacial electrical potential which we suggest generates an additional solvation contribution to the total free energy that is traditionally overlooked in continuum descriptions of interparticle interactions in solution. In the present investigation we perform molecular dynamics simulation based calculations of the interfacial potential using realistic surface models representing various chemistries as well as different solvents. Similar to previous work that focused on simple model surfaces constructed by using oxygen atoms, we find that solvents at more realistic model surfaces exhibit substantial anisotropic orientational behavior. We explore the dependence of the interfacial solvation potential on surface properties such as surface group chemistry and group density at silica and carboxylated polystyrene interfaces. For water, we note surprisingly good agreement between results obtained for a simple O-atom wall and more complex surface models, suggesting a general qualitative consistency of the interfacial solvation effect for surfaces in contact with water. In contrast, for an aprotic solvent such as DMSO, surface chemistry appears to exert a stronger influence on the sign and magnitude of the interfacial solvation potential. The study carries broad implications for molecular-scale interactions and may find relevance in explaining a range of phenomena in soft-matter physics and cell biology.

## Introduction

A molecular-level description of the interface of an electrolyte with a solid surface or a gas has greatly enhanced our understanding of a wide range of phenomena such as the surface tension of water,^1,2^ ion adsorption,^3^ electrochemical energy conversion,^4^ and electrokinetic effects.^5,6^ A powerful predictive tool, molecular dynamics (MD) studies, able to probe dynamics on the nanosecond time scale, have shed light on the origin of vibrational spectroscopy signatures of interfacial water,^1,7^ the short-range hydration forces between biological membranes,^8^ and nanopore gating mechanisms.^9,10^ Interfacial water has also been implicated in protein aggregation,^11^ the thermodynamics of molecular binding interactions,^12,13^ and various cellular functions.^14,15^ However, despite recent successes of both simulation and theoretical approaches, a complete picture of the molecular-level organization of solvent molecules and ions at the interface is often lacking for many challenging problems, for example, when the process being studied involves chemical reaction pathways or takes place under nonequilibrium conditions.^14−16^ Given the critical importance of interfacial water to thermodynamic properties of soft matter and liquid state systems, much effort is currently being devoted both to the development of improved descriptions for intermolecular and water–ion interactions^17,18^ and to the study of molecular water in contact with a diverse array of substrates.^15^

Recently, we proposed a model for a long-ranged force due to interfacial solvation, in the context of interparticle and intermolecular interactions in solution. In this picture, the orientational response of interfacial water molecules at an electrically charged surface contributes an excess free energy which is absent in traditional models that treat water as a continuum.^19,20^ Because the magnitude of this contribution can be substantial, and because of its potentially overarching importance in the understanding of intermolecular interactions in solution, this study examines in detail the orientational behavior of water and solvents such as dimethyl sulfoxide (DMSO) as a function of surface chemistry.

**Figure 1:**
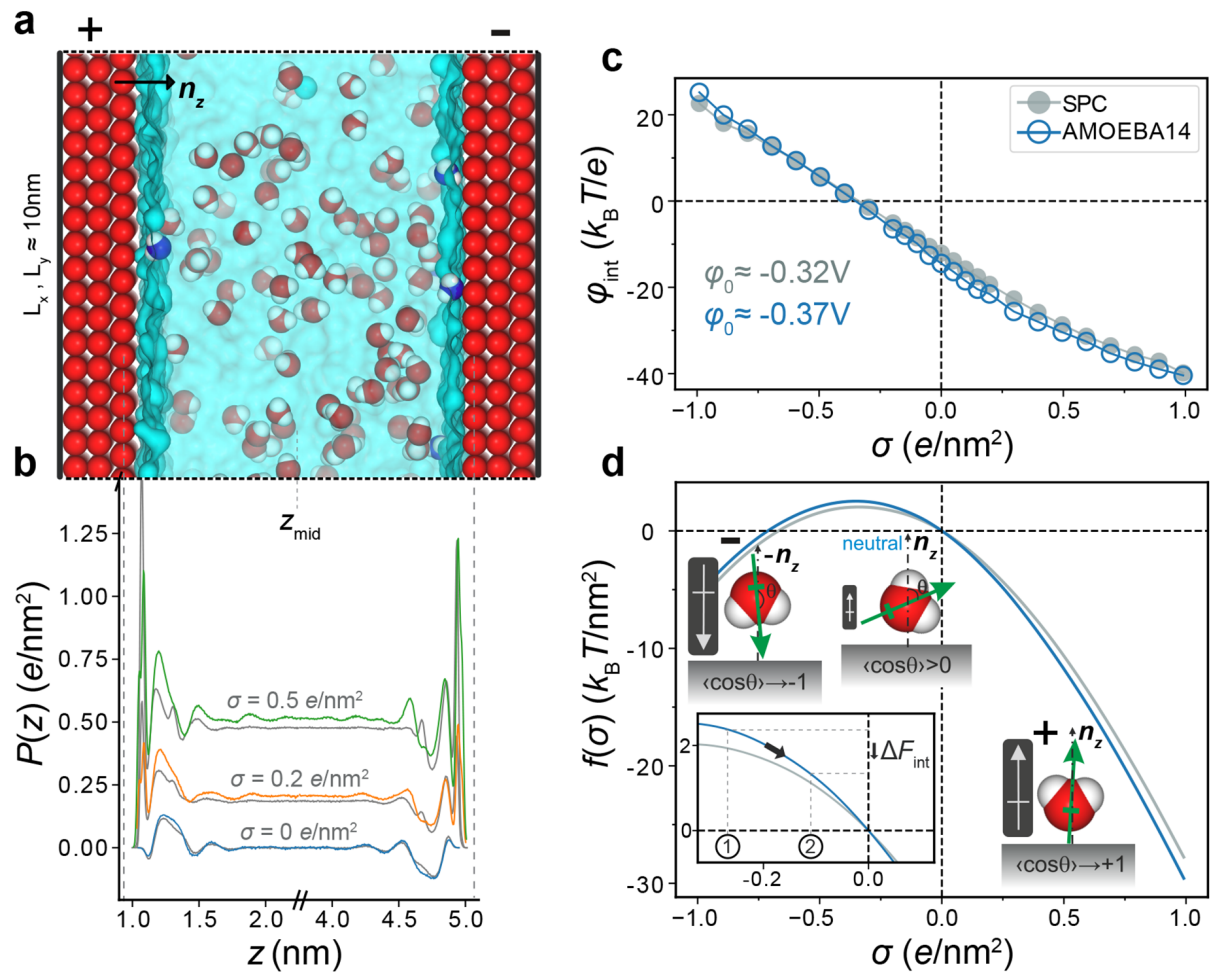
Excess hydration free energy of a charged interface for the SPC and AMOEBA14 water models. (a) Schematic representation of the capacitor simulation cell, consisting of water molecules confined between two plates of dimensions ≈10 × 10 nm^2^, each made up of three layers of hexagonally packed, positionally restrained oxygen atoms, ≈4 nm apart along the *z*-direction. A subset of the interfacial wall atoms on the left and right plates are randomly assigned integer charge of ±1*e* respectively, to generate an overall charge density of ±σ. (b) Area-averaged polarization profiles, *P*(*z*), across the simulation box, extracted from the MD simulations. The AMOEBA14 results (colored curves) are overlaid onto the SPC water model results (gray curves). Note that the projection of **μ** along *z*, μ_*z*_, corresponds to a projection along the surface normal **n**_***z***_ directed as shown in (a). (c) The excess electrical potential at the walls, φ_int_(σ), is derived from *P*(*z*) by integration from the reference position, *z*_mid_, as described in the text. (d) The excess hydration free energy per unit area, *f*(σ), obtained by integration of φ_int_(σ), as described in the main text. The nonmonotonic trend in interfacial free energy arises from a net orientation of the water molecule dipole at a neutral O-atom surface, which then flips direction as the surface becomes more negatively charged, as depicted in the water molecule schematics. In an interaction between two approaching negatively charged particles, the transition from state 1 to state 2 (shown in the inset) corresponds to a decrease in interparticle separation which is accompanied by a reduction in surface charge density, σ, due to charge regulation. A decrease in σ is coupled with a reduction in interfacial free energy, Δ*F*_int_, which counteracts and can even dominate the electrostatic repulsion Δ*F*_el_ at long range.^19,20^

At the interface of a pure ion-free liquid with a vapor, interfacial solvent molecules display a net broken orientational symmetry relative to the bulk, which gives rise to an excess solvation potential or interfacial potential. This quantity is not only of importance to interparticle interactions but also highly relevant in the field of electrochemistry.^21−24^ In water specifically, the broken orientational symmetry at an interface is believed to arise from the bent-core structure of the molecule and its ability to preferentially hydrogen bond toward the bulk. At a neutral interface, water orients such that the O atoms on average point slightly toward the interface.^19,25−28^ A similar anisotropy in orientation has also been implicated in the asymmetric solvation of ions, where, for example, water preferentially solvates anions compared to cations.^25,29−31^ In this investigation we focus on the excess interfacial potential, φ_int_, which represents the portion of the electrical potential due to molecular orientation at an interface that is not accounted for within a continuum electrostatic description. At an electrically charged interface, this excess interfacial potential due to the solvent gives rise to an excess solvation free energy which does not appear in continuum descriptions of the electrostatic interaction between charged objects in solution and yet must make a contribution to the overall interaction free energy.^19,20^ The excess free energy for water at highly charged surfaces has been found to be large, negative, and monotonically increasing as a function of charge density, regardless of the sign of charge at the surface^19,32^ (Figure 1). But importantly, we have found that for water the excess free energy exhibits pronounced nonmonotonic behavior for surfaces carrying low values of negative surface charge density (|σ| < 0.3 *e*/nm^2^).^19^ This charge-asymmetric behavior of the excess free energy arises from the fact that water molecules at an uncharged surface display a small amount of net orientation, which gives rise to a negative value of the excess interfacial potential at the surface (i.e., φ_0_ = φ_int_(σ=0) < 0) and has profound consequences for interparticle interactions in solution, as described below.^19,33^

To provide a broader context for this investigation, we briefly summarize our model for interparticle interactions in solution that incorporates an excess free energy contribution due to the interfacial solvent at an electrically charged surface.^19,20^ The model combines the electrostatic free energy from the Poisson–Boltzmann (PB) equation with an interfacial free energy contribution arising from the orientational anisotropy of the solvent at an interface. This model can explain key features of the experimental observations of like-charge attraction between colloidal particles in solution.^34,35^ Such experimental observations have long evaded theoretical explanation.^35−37^ Calculations of mean-field PB free energies applied within the context of the Derjaguin–Landau–Verwey–Overbeek (DLVO) theory alone are unable to account for a stable minimum in the interaction potential between two like charged objects at long range (5κ^–1^–10κ^–1^) and in solutions of low ionic strength (<0.1 mM).^36,38,39^ Here κ^–1^ denotes the Debye length, which represents the rate at which the electrical potential at the surface of a charged object in solution decays as a function of distance. We previously demonstrated agreement between the model and the experimental data for different sets of experiments, covering an order of magnitude in particle size and a broad range of experimental conditions.^19,20^ We found that under specific conditions determined by the pH and ionic strength in solution, the p*K* of the ionizable surface groups, and their number density, our calculations of distance-dependent interaction free energies for a pair of particles in solution revealed a long-ranged minimum at *x* ≈ 5κ^–1^, where *x* is the intersurface separation between the two spheres. Thus, at long-range , calculations for large spheres (*R* ≫ κ^–1^) revealed a total interaction potential given by the sum of two terms, of the form
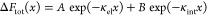
1with *A* > 0.^19,20^ Here, the first term represents the overall repulsive electrical free energy, Δ*F*_el_(*x*) = *A* exp(−κ_el_*x*), arising from the PB free energy, and the second term, Δ*F*_int_(*x*) = *B* exp(−κ_int_*x*) denotes the free energy contribution from interfacial solvation. Note that κ_int_ < κ_el_ ≈ κ^–1^.^19,20^ Importantly, the Δ*F*_int_(*x*) term implies an attractive contribution to Δ*F*_tot_ when *B* ∝ φ_int_(σ) < 0.^20^ The excess interfacial potential for water an interface is a function of the charge density, σ, of the surface and may be written as φ_int_(σ) = φ_0_ + *k*σ, where φ_0_ ≈ −0.3 V < 0 for the simple point charge (SPC) water model calculated at an oxygen atom wall.^19^ At low values of σ we have φ_int_(σ → 0) ≈ φ_0_, which gives *B* ∝ φ_0_. Therefore, the net orientation of water molecules, manifesting in the sign of the excess interfacial potential φ_0_ at an uncharged surface, generally determines the sign of the interfacial contribution Δ*F*_int_ to the total free energy of interaction.

Thus, in summary, two approaching like-charged particles in general experience a progressive reduction in the magnitude of their surface charge due to a phenomenon known as charge regulation.^40−42^ The interfacial free energy per unit area for a surface with a charge density σ is then simply obtained by using the charging integral , which yields the function *f*(σ) = φ_0_σ + *k*σ^2^/2, which in the limit of low charge density may be written as *f*(σ) ≈ φ_0_σ. The total interfacial interaction energy is then given by an integral of *f*(σ) over the particle surface, *F*_int_(*x*) = *∫*_*S*_*f*(σ;*x*) d*S*. We find that referenced to its value at infinite interparticle separation (*x* → *∞*) the integral of *f*(σ) over the particle surfaces gives an interfacial free energy contribution Δ*F*_int_(*x*) that increases exponentially in magnitude with decreasing separation *x*, as shown in eq 1.^19,20^ Importantly, Δ*F*_int_(*x*) < 0 implies an attractive contribution to the total free energy which mitigates the electrostatic repulsion, whereas Δ*F*_int_(*x*) > 0 implies a repulsive contribution to the total free energy that augments the nominal electrostatic repulsion. Thus, the value and sign of φ_0_ are key in explaining both qualitative and quantitative aspects of the experimental observations. The qualitative aspects refer to the fact that, in water, negative particles are expected to attract, while positives repel, and the quantitative features concern the depth and location of the interparticle potential minimum in an interaction between negatively charged particles.^19,20^ In summary, our PB model incorporating interfacial solvation effects indicates that weakly charged negative particles in aqueous solution may attract rather than repel at long range. For positively charged surfaces, on the other hand, we expect that the canonical like-charged electrostatic repulsion is reinforced by the excess free energy contribution from the interfacial solvent, which is consistent with experimental observations.^34,43,44^ Note that, in addition, the model suggests that the interfacial contribution should be repulsive for strongly negatively charged (|σ| > 0.3 *e*/nm^2^) surfaces, in contrast to weakly negatively charged surfaces.

The nonmonotonic trend in interfacial free energy shown in Figure 1d relies on a negative value of the excess interfacial potential at an uncharged surface, i.e., φ_int_(σ = 0) = φ_0_ < 0. In particular, at the interface of an O-atom wall with water we have φ_0_ ≈ −0.3 V calculated relative to a point in the *bulk liquid*.^19^ This arises from the slight preferential orientation of negative O atoms toward the interface as shown in Figure 1d. Referencing the interfacial potential relative to the wall interior rather than the bulk liquid would give an interfacial potential of opposite sign, namely ca. φ_0_ ≈ +0.3 V. Indeed, some indirect electrochemical estimates of the interfacial potential place its value at about +0.1 V with respect to a vacuum, which is in qualitative agreement with our calculated value of approximately +0.3 V (and +0.2 V for the vapor/water interface) for the SPC water model.^24,45^ We note that the positive sign agrees with the potential of about +3.5 V calculated from the quantum mechanical charge distribution using density functional theory (DFT).^22^ The value of +3.5 V from DFT-MD lies in close agreement with electron holography measurements of vitrified ice,^46^ capable of probing the interior electrostatic potential of water molecules.^22,23^ However, the DFT value includes a contribution from the quadrupole moment trace of the molecular charge distribution. It is well-known that unrestricted spatial averaging of the electrical potential in DFT may not reflect the value of the electrical potential felt by ions and ionized groups that reside in the interstitial spaces between water molecules in solution.^22,23,45^

We emphasize that to calculate the surface potential relevant to electrochemistry from classical MD simulations, we consider only the dipolar and traceless quadrupolar contribution of the modeled molecular charge distribution to the interfacial potential.^23^ The traceless quadrupole moment density is zero in the bulk liquid, where solvent molecules are randomly oriented, and tends to zero at the interface with the solid phase, where the density of solvent molecules vanishes. Therefore, evaluation of the contribution to the interfacial potential φ_int_ due to the traceless quadrupole density is approximately zero, and only the dipolar term remains (see the Supporting Information, section 1).^23^ We point out that inclusion of the *nontraceless* quadrupolar moment of the SPC water model changes the sign of the calculated interfacial potential to about −0.5 V with respect to a vacuum^21,47^ or +0.5 V with respect to bulk for comparison with our work. However, as described above, the potential of interest in our work is the potential outside the molecular envelope which should not contain a contribution from the quadrupole moment trace of the molecular charge distribution.^22,23,45^

There is an additional caveat in the calculation of surface potentials from classical MD simulations as outlined above, which arises from the fact that the potential due to the dipole contribution appears to be dependent on the choice of a molecular center used to locate the molecular dipole moments. This is a well-known issue which has been discussed in depth previously and can be understood simply as a consequence of the way in which molecules are partitioned between regions of space when employing a molecular based cutoff (M-scheme).^48−53^ In this study, we consider the oxygen atom as the molecular center for SPC water, which is the sole van der Waals (vdW) site of the molecular model.^50^ For molecules with more than one vdW site, we generate an interfacial population of molecules for the calculation of the interfacial potential using the identification of truly interfacial molecules (ITIM) algorithm (see the Supporting Information, section 1, for further details).

In previous work,^19^ we extracted values for φ_int_(σ) by simulating the behavior of SPC water at a wall that only supported Lennard-Jones (LJ) interations and was composed of positionally restrained oxygen atoms that were randomly assigned integer charges, corresponding to a total surface charge density σ. In this study, we first extend the original work by considering a more sophisticated water model, AMOEBA14, which is a three-site polarizable water model. This enables us to examine the effect of polarization on the calculated interfacial potential and excess solvation free energy. The AMOEBA14 model allows water molecules to adapt their dynamics to their local environment via induced dipoles on each atomic site. Interestingly, we find that a more complex water model does not yield a φ_int_(σ) trend that is significantly different from that of SPC water (Figure 1). Therefore, we revert to the use of the simpler and more computationally efficient SPC water model in subsequent simulations in the study. We further introduce an added level of realism to the molecular model of the interface by replacing the LJ O-atom walls with silica and carboxylated polystyrene surfaces that constitute the majority of the experimental like-charge attraction observations.^35,54−56^

Finally, we draw upon asymmetric solvation studies at ion sized cavities^25^ to select a solvent, namely DMSO, that is expected to display the opposite orientational asymmetry at an interface compared to molecular water,^25^ implying φ_0_ > 0. We find, however, that while the calculated excess interfacial potential, φ_0_, for DMSO is about +0.3 V at a model O-atom surface, it can in fact be large and negative for DMSO in contact with silica surfaces. This observation highlights the possible impact of surface chemistry on the value of the excess interfacial potential in specific solvents.

## Simulation Methods and Analyses

This section discusses in detail the methods and procedures used to carry out the simulations and subsequent analyses in this work. Further details concerning system preparation and simulation settings can be found in the Supporting Information. Example input files, force field parameters, and code for the analysis of the simulations performed in this study are available on our GitHub page: https://github.com/rowanwalker96/interfacial̇potential.

### Molecular Dynamics in a Capacitor Setup

In our simulations we generally calculate the excess interfacial potential φ_int_(σ) as a function of surface charge density σ by using a parallel-plate capacitor system wherein a slab of solvent is sandwiched by model solid surfaces carrying variable amounts of net electrical charge, as described previously.^19^ An exception concerns silica surfaces in contact with DMSO and polystyrene surfaces, where we only calculate the value of φ_0_ and therefore use a system consisting of a single surface in contact with solvent. The main advantage of the capacitor system is that it enables simulations involving charged surfaces where electroneutrality in the simulation box is ensured without having to include charge-compensating ions in solution. In addition, the system also simultaneously yields estimates of φ_int_(σ) for both positive and negative values of σ and provides a well-defined system for comparing solvation at a macroscopic surface with a continuum electrostatics model.

We first study different water models enclosed between capacitor plates composed of positionally restrained oxygen atoms that only support LJ interactions. The plates are ≈10 × 10 nm^2^ in area and are separated by ≈4 nm of solution in the *z*-direction (Figure 1a). A subset of the atoms belonging to the first layer in each wall (in direct contact with the solvent) is randomly assigned a positive (left plate) or a negative charge (right plate) to generate an electric field of specific strength in the box. In studies of the interface between a silica surface and water, we replace the negatively charged O-atom plate located at *z* ≈ 5 nm with a silica slab capable of acquiring a negative surface charge, σ < 0, via deprotonation of surface silanol groups. Finally, in simulations that examine the influence of the solvent species on the value of the interfacial potential we replace the water in the capacitor setup with the solvent of interest, DMSO in this study.

### Implementation of the AMOEBA14 Polarizable Water Model

Here we discuss the use of the AMOEBA14 polarizable water model^17^ to compute the interfacial potential at an LJ O-atom wall. To enable a direct comparison between the results for the AMOEBA14 model and the simpler SPC model, we employ our simple model capacitor system, consisting of 12448 water molecules between two confining walls of positionally restrained oxygen atoms with the same LJ parameters as the water oxygens (Figure 1a).^19^ The calculations using the AMOEBA force field were performed with the molecular modeling package OpenMM.^57^ The molecular dynamics (MD) simulations were performed in the NVT ensemble at 300 K, with temperature maintained via a Langevin thermostat with a coupling constant of 0.1 ps. The particle mesh Ewald (PME) method was used to evaluate the electrostatic interactions with 3D periodic boundaries. We introduced a vacuum layer of twice the slab length in the *z*-direction and applied the 3dc correction of Yeh and Berkowitz^58,59^ to remove artificial polarization induced by neighboring image dipoles (see the Supporting Information, section 2, for further details).

The overall dipole moment of a water molecule described by the AMOEBA14 model has three contributions: the dipole generated by the atomic partial charges, the permanent atomic dipoles, and the induced atomic dipoles. We binned the dipole generated by the atomic partial charges on the oxygen atom coordinate of the water molecules (as was done for SPC water). We binned the vector sum of the permanent and induced dipoles, which are located on every atomic site, on the center of geometry of the water molecules. We noted that a change in the binning location of the molecular dipole moments can yield slightly different results in the calculation of the interfacial potential φ_int_ (see the Supporting Information, section 2, for further details). Simulations implemented full mutually induced polarization and the induced dipole calculation was iterated until convergence. The systems were equilibrated for 100 ps followed by production simulations of 1 ns, with trajectory frames written every 0.1 ps.

### Modeling Water at an Interface with a Silica Surface

**Figure 2:**
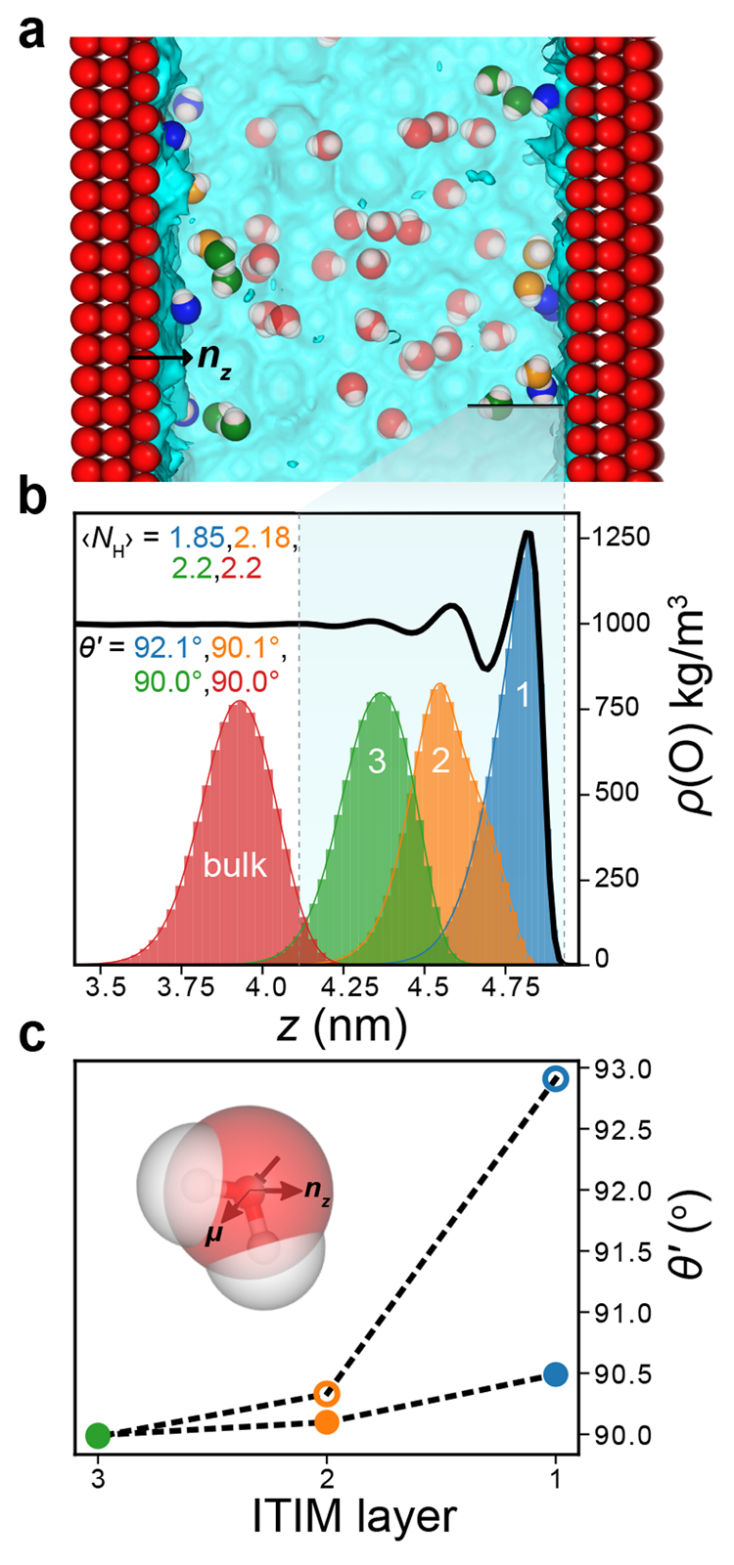
ITIM and hydrogen bond analysis of SPC water at an uncharged O-atom surface. (a) Simulation snapshot illustrating the various types of interfacial water molecules identified by using the ITIM algorithm, emphasizing a first interfacial layer (blue) and subsequent layers (orange, green). (b) Density distributions of water molecules for each ITIM layer (blue, orange, and green) compared to a layer situated in the bulk (red). The peak observed in the total water density (black curve) arises largely from molecules in the first layer (blue). Values for the average number of hydrogen bonds formed per water molecule, ⟨*N*_H_⟩, are quoted for each ITIM layer. Also presented are angles θ′ (θ′ = cos^–1^(⟨**μ̂**⟩·**n**_*z*_)), showing that water molecules in first layer point their hydrogen atoms away from the wall. (c) Angle θ′, calculated for subsets of water molecules that act as either hydrogen bond donors (open circles) or acceptors (filled circles), for each ITIM layer. The orientational anisotropy in the first ITIM layer largely arises from hydrogen bond donors in that layer that preferentially hydrogen bond toward the bulk liquid.

**Figure 3:**
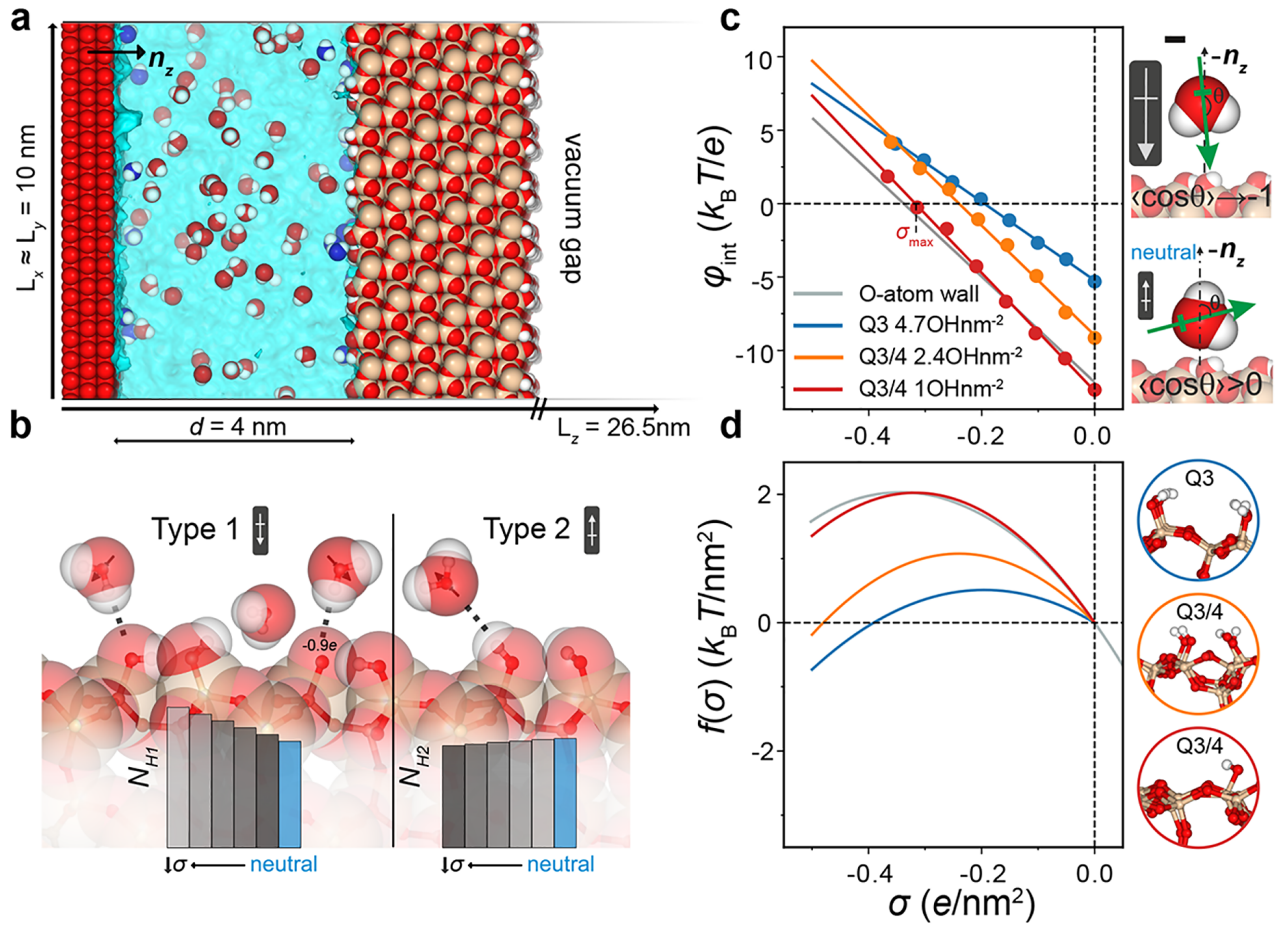
Free energy of surface solvation at the silica–water interface. (a) Schematic representation of the cross section of a simulation cell. We use a parallel plate capacitor setup, with the negative O-atom plate replaced by a silica slab. The system dimensions are ≈10 × 10 × 8.5 nm^3^ with a large vacuum gap left in the *z*-dimension—required to apply the 3dc correction to the Ewald sum for the correct treatment of long-range electrostatics. Periodic boundaries apply in all three dimensions. Deprotonation of a silanol group results in a negative surface charge of −1*e* on the silica surface. This charge is balanced by assigning +1*e* to O atoms of the opposing wall in the capacitor setup. The gap between the two surfaces is filled with ≈12500 SPC water molecules. (b) Illustration of the two types of hydrogen bonds formed between the silica surface and interfacial water molecules. Type 1: the oxygen atoms of the surface silanol groups act as hydrogen bond acceptors. The dipole orientation of the water molecules involved in type 1 hydrogen bonds is largely in the positive *z*-direction in our system (oriented toward the silica surface). The siloxane bridge oxygen is also found to be a weak hydrogen bond acceptor, and this contribution is included within the type 1 category. Type 2: the oxygen atoms of the surface silanol groups act as a hydrogen bond donors. The dipole orientation of water molecules involved in type 2 hydrogen bonds is opposite to that of the type 1 molecules, pointing on average in the negative *z*-direction (away from the silica surface). Also shown are the number of type 1 and 2 hydrogen bonds *N*_H1_ and *N*_H2_ for variable surface charge densities σ. (c) Excess interfacial potential, φ_int_(σ), determined for silica surfaces of varying silanol group density (4.7 OH nm^–2^, blue; 2.4 OH nm^–2^, orange; 1 OH nm^–2^, red) compared with the result for water at an O-atom surface (gray line). Schematics on the right display net water molecule orientation at a neutral silica surface (with the O atom pointing toward the surface) and at a strongly negatively charged surface where an inversion of the net water molecule dipole occurs. (d) Plots of the excess hydration free energy per unit area, *f*(σ) vs σ, obtained from integration of the data in (c). As silanol group density decreases, *f*(σ) approaches that of water at a simple O-atom LJ surface (gray curve).

To model solvation at a silica interface, we use a capacitor setup similar to that in the previous section, replacing the negatively charged O-atom plate in the capacitor with a silica surface. The opposing wall in the capacitor setup remains unaltered, i.e., composed of oxygen atoms that may be assigned a positive charge (Figure 3a). Starting silica structures derived from the 101̅ cleavage plane of α-cristobalite were generated with the CHARMM-GUI Nanomaterial Modeler platform.^60^ We considered surfaces with various surface silanol group densities ranging from types Q3 (4.7 OH per nm^2^) to Q4 (0 OH per nm^2^), for which the surface silicon atoms have either one or no attached silanol groups respectively, as shown in Figure 2. To a first approximation, Q3 surfaces are representative of silica glasses.^61^ Surfaces with intermediate silanol group densities (Q3/Q4) are constructed by using a mixture of Q3 and Q4 environments. Surfaces with a lower density of silanol groups (<4.7 OH per nm^2^) are used to model heat-treated silica surfaces and nanoparticles.^61^ We employed the INTERFACE force field, which has been parametrized to model charged silica–water interfaces and shown to accurately reproduce key interfacial properties such as water contact angles, water adsorption isotherms, and adsorption energies of peptides.^61,62^

Prior to performing simulations with the “hybrid” capacitor setup described above, we first ran short preliminary simulations of a single solvated silica slab at constant atmospheric pressure. We then extracted the equilibrated configuration of the silica wall and introduced it into our capacitor setup, at the location of the nominally negatively charged LJ wall (right wall at *z* ≈ 5 nm in our convention; see Figure 3a). We then randomly deprotonated some surface silanol groups to generate systems with a given negative surface charge density, σ, and updated the topology of the surface atoms accordingly. The surface of the opposing oxygen atom wall was assigned an opposite positive charge of identical overall magnitude. Periodic bonds across simulation cell boundaries in the periodic *xy*-directions were generated, and the heavy atoms of the silica slab were positionally restrained with a force constant of 400 kJ mol^–1^ nm^–2^ to keep the surface rigid. SPC water molecules were then introduced into the capacitor setup. The particle mesh Ewald (PME) method was used to evaluate the long-range electrostatic interactions with 3D periodic boundaries by using a 1 Å grid spacing and a short-range cutoff of 12 Å. The LJ interactions were smoothed over the range of 10–12 Å by using the force-based switching function. A large vacuum gap was left in the *z*-direction (Figure 3a) and the 3dc correction applied to the Ewald sum. MD simulations were performed by using the GROMACSv2019.4 MD code^63^ in the NVT ensemble at 300 K. Systems were energy minimized and then equilibrated during 500 ps in the NVT ensemble by using a V-rescale thermostat at 300 K with a coupling constant of 0.1 ps. Following this, production simulations were performed for a total of 5 ns, with the temperature now maintained via a Nosé–Hoover thermostat with a 1 ps coupling constant. Constraints were applied with the LINCS algorithm to all bonds involving hydrogen atoms in the system.^64^ Center-of-mass motion removal was applied, and the trajectory frames were written every 0.2 ps.

### Modeling and Analysis of Interfacial Properties at a Carboxylated Polystyrene–Water Interface

Initial polystyrene melts were generated by using the CHARMM-GUI polymer builder^65^ via a coarse-grained model simulation of 117 chains of atactic polystyrene, each consisting of 32 monomers, at 425 K. The resulting structure was then converted to an all-atom structure. Further system preparation was then performed with GROMACS and relied on simulated annealing from 400 to 425 K followed by cooling to 300 K at a rate of 0.01 K/ps, all under constant pressure maintained via a Berendsen barostat. The polystyrene melt prepared in this way has an area of ≈9.5 × 9.5 nm^2^, adopts slab geometry in the *xy*-plane, and reproduces the experimental density of ≈1 kg/m^3^. We quantified the surface roughness as described in the Supporting Information and found that the values were in agreement with previous simulation work and atomic force microscopy (AFM) measurements of spin-coated polystyrene.^66^

**Figure 4:**
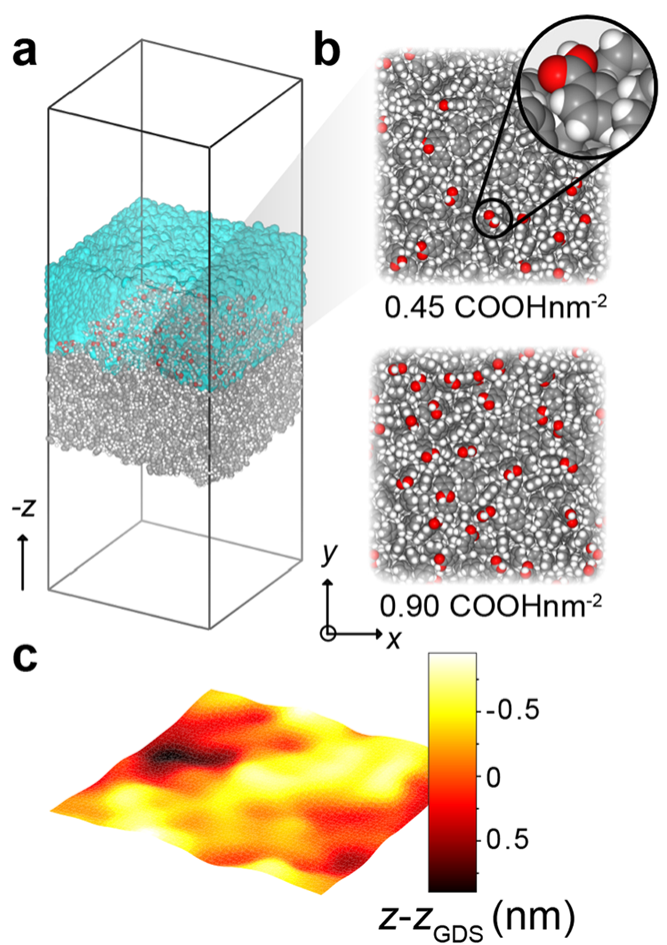
Model systems of carboxylated atactic polystyrene (PS). (a) Graphical representation of a simulation cell for a solvated PS system. Cell dimensions measure ≈10 × 10 × 30 nm^3^. (b) Carboxyl groups are randomly attached to the ortho and meta positions of surface styrene rings in a surface region of ≈1 nm in depth. The surface carboxyl group densities considered here are 0.45 and 0.9 nm^–2^. (c) Surface topography of a PS surface in contact with SPC water molecules in the MD simulations. The heat map depicts the instantaneous molecular interface which clearly displays an uneven topography due to surface roughness over an interfacial region of width ≈2 nm in *z*. Surface heights presented here are calculated with respect to the Gibbs dividing surface (see the Supporting Information, section 5) of the PS surface.

Carboxyl surface groups were attached in the ortho and meta positions of randomly selected styrene rings in the surface region (of ≈1 nm depth), while ensuring that this generated no major steric clashes. The force field parameters for the polystyrene and attached carboxyl groups were taken from the CHARMM generalized force field, CGenFF.^67^ Systems were then energy minimized and solvated with the SPC water model prior to preliminary equilibration simulations of 5 ns in the NPT ensemble with the Parrinello–Rahman pressure coupling method. The final structure was then used as a starting configuration for production simulations lasting 10 ns in the NVT ensemble at 300 K. The temperature was maintained with a Nosé–Hoover thermostat with a coupling constant of 0.1 ps. Water molecules were present only on one side of the polystyrene (PS) slab and a vacuum gap of twice the resulting total slab thickness left above in *z* so as to apply the 3dc correction (Figure 4a).

### Quantification of the Excess Solvation Free Energy

To quantify the excess solvation free energy at an interface, we require the axial polarization *P*(*z*) as a function of *z*, calculated in the capacitor setup. *P*(*z*) is calculated as *P*(*z*) = *P*_1_(*z*) – ∇*Q*_*zz*_(*z*) where *P*_1_(*z*) is the dipole moment density, ρ(*z*)μ_*z*_(z), and *Q*_*zz*_(*z*) is the traceless quadrupole moment density^23^ (see the Supporting Information, section 1, for further details). We found that the polarization at the midplane of the capacitor, *P*(*z*_mid_), calculated from the MD simulations, agrees well with the value of the polarization, , expected for a capacitor with continuum water as the dielectric material of relative permittivity ϵ. At an interface, however, we note that *P*(*z*) in the capacitor departs substantially from the continuum value. This is due to symmetry breaking in the orientational behavior of the solvent induced by the presence of an interface.

Integrating *P*(*z*) from the reference position at the midplane of the capacitor, *z*_mid_, up to the surface of each plate, gives the total electrical potential at the plate due to solvent polarization. Subtraction of the integral of the polarization for a continuum-dielectric capacitor from that of the simulated polarization gives an estimate of the “excess” interfacial potential, φ_int_. Thus, we evaluate φ_int_ as
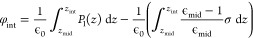
2where the first term is the potential calculated from MD, by integration of the dipole moment density *P*_1_(*z*), as outlined in ref (23), and ϵ_0_ is the permittivity of free space. The contribution of the traceless quadrupole moment density *Q*_*zz*_(*z*) to the interfacial potential is approximately zero, and for this reason we do not include it in the evaluation (see the Supporting Information, section 1, for further details). The second term is the estimated electrical potential contribution from the continuum electrostatics model where the water in the capacitor is regarded as a featureless dielectric continuum. Importantly, ϵ_mid_ is not exactly 80 but is set to the value of ϵ at the midplane of the capacitor, as calculated from the expression . In turn, *z*_int_ denotes an interfacial plane which for an O-atom wall we define to be located where *P*_1_(*z*) finally drops below the continuum value, as in previous work.^19^

Unlike an O-atom wall, our silica surfaces display a small amount of surface roughness which gives rise to an interfacial region that contains both surface atoms and interfacial solvent molecules. We therefore construct a continuum electrostatics model at the silica–water interface in a slightly different way and evaluate φ_int_ as
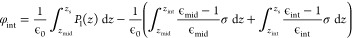
3where *z*_int_ is now defined to be located where the water density ρ(*z*) finally falls below the bulk value. This choice of location coincides with the onset of a nonzero density for the surface atoms. The coordinate *z*_int_ therefore delimits the bulk region where ϵ = ϵ_mid_ from the “interfacial zone” (*z*_int_ < *z* < *z*_s_) where we set ϵ = ϵ_int_ = 1. *z*_s_ denotes the surface phase, where the local density of water molecules ρ(*z*) is zero. Setting ϵ_int_ = 1 is expected to capture the effectively nonpolarizable or weakly polarizable nature of the interfacial zone.^47^ We point out that changing the value of ϵ_int_ does not have a substantial influence on the final results (see the Supporting Information, section 4, for further details). The difference between the electrical potentials derived from the simulation and a continuum model (first and second terms in eq 3) gives the “excess” potential φ_int_. We repeat this calculation for simulations in the capacitor setup with variable values of surface charge σ and extract a profile for φ_int_(σ). Finally, we calculate a charging integral of the form , which gives the excess hydration free energy per unit area of surface, which arises from the excess polarization of water molecules at an interface.

We note that the location of *z*_int_ is not unique, and the definition of this interfacial plane can have an impact the final integrated value of φ_int_. Our choice here has been to define *z*_int_ by using a criterion based on water density as described above. Importantly, *z*_int_ only requires to be defined in systems with charged surfaces, where an estimate of the “excess” potential, φ_int_(σ ≠ 0), from the simulated polarization requires a corresponding reference value from continuum electrostatics. For uncharged surfaces, the calculation of φ_0_ does not involve subtraction of a continuum electrostatics contribution which is implicitly zero. Equation 3 therefore reduces to the first term, and we evaluate the first integral to a distance of *z*_s_ to determine φ_0_. Furthermore, in the regime of low to moderate surface charge densities that is of interest in most experimental situations (σ ≲ 0.1*e* nm^–2^), the excess hydration free energy can be well approximated as *f*(σ) ≈ φ_0_σ. This renders comparisons of simulation results with experimental data at low surface charges densities robust to the choice of *z*_int_ (see the Supporting Information, section 4, for further details).

### Classification of Hydrogen Bonds at a Solvated Interface

In our simulations, we identify a hydrogen bond based on geometric criteria given by a donor–acceptor distance of less than 3 Å and a donor–H–acceptor angle of over 150°.^69^ For silica, the surface groups are amphoteric in nature; i.e., the surface silanol oxygens can act both as hydrogen bond donors (type 2) and acceptors (type 1) (Figure 2b).^70,71^ The siloxane bridge oxygen is also found to be a weak hydrogen bond acceptor,^26^ and this contribution is included within the type 1 category. We use the same convention for classifying hydrogen bonds between the carboxyl groups on our polystyrene surfaces and interfacial water molecules, with the carboxyl O atoms behaving as either hydrogen bond donors (type 2) or acceptors (type 1).

### Analysis of Interfacial Properties with the ITIM Algorithm

We employ the identification of truly interfacial molecules (ITIM) algorithm,^72^ implemented in the Pytim package,^73^ in situations where we wish to analyze the properties of water molecules that belong to distinct hydration layers at a surface or to effectively remove the surface roughness in the calculation of interfacial properties. The algorithm involves moving probe spheres of a given radius along streamlines that lie perpendicular to the surface of interest and that are separated by some grid spacing. When the vdW sphere of an interfacial solvent atom is hit by the probe sphere, the molecule to which that atom belongs is labeled as an interfacial molecule and assigned a layer number corresponding to the order in which it was encountered along the streamline.^72^ In this way a layer-by-layer molecular representation of the interface can be constructed. For all applications of the ITIM algorithm in this study we used a mesh spacing of 0.2 Å, a probe radius of either 1.5 or 2 Å for simulations involving water and DMSO, respectively, and input the vdW radii for the simulated atom types defined by the corresponding force field in each case.

## Results and Discussion

### Comparison of the Excess Interfacial Potential between the SPC and AMOEBA14 Water Models at a Model O-Atom Surface

The polarization profiles, *P*(*z*), of SPC water and AMOEBA14 water models calculated along the *z*-direction of the capacitor are in close agreement with each other for water in contact with an O-atom surface (Figure 1b). As a result, the curves for the excess free energy of interfacial solvation, *f*(σ), calculated by using the two water models are also very similar (Figure 1d). In the weakly negatively charged regime (|σ| < 0.3 *e*/nm^2^), we note that the two curves are virtually identical. We emphasize, however, that different values of the interfacial potential can be obtained for a different choice of molecular center used to locate the molecular dipole moments but that the sign of the interfacial potential is consistent (see the Supporting Information, section 2).

The AMOEBA14 model differs from the SPC model in that it includes induced dipoles that respond to their local environment. Examination of the *z*-component of the induced dipoles in the system reveals that they in fact only produce a small enhancement of the net local dipole density (i.e., they reinforce slightly the molecular dipoles due to the atomic charges and to the permanent atomic dipoles). The *P*(*z*) profiles between the two water models also differ slightly in the values of the permittivity in the bulk liquid. We further note that switching on the polarizability of the uncharged capacitor wall atoms does not significantly alter our results. However, the polarizability of the surface may be expected to play a more prominent role at surfaces that host charged polar groups or when in contact with a concentrated electrolyte.^74,75^

Thus, it appears that the SPC water model captures well the orientational behavior of interfacial water molecules when compared to a physically more sophisticated yet more computationally expensive water model. Comparing with other water models, for the TIP3P water model—another planar (2D), nonpolarizable water model—we obtain a value for φ_0_ ≈ −0.3 V which is very similar to the SPC water model. It is also worth noting that 3D water models, where the molecular charge distribution has 3D character, generally yield values of φ_0_ at the water–vapor interface that are of the same sign but of smaller magnitude than the results for the 2D model counterparts.^23^

### ITIM and Hydrogen Bond Analysis at an O-Atom Interface

Here we present a detailed analysis of the orientational behavior of SPC water at a simple O-atom surface. We spatially segment the system in the vicinity of the capacitor walls into distinct molecular layers using the ITIM algorithm.^72^ We then examine properties such as the average dipole moment and hydrogen bonding orientations for each molecular layer over the simulation trajectories.

We find that the first layer of interfacial water molecules (i.e., closest to the wall) provides the dominant contribution to the largest peak in the water density profile (Figure 2b). Water molecules in this “first hydration layer” are on average involved in 1.85 hydrogen bonds per molecule (⟨*N*_H_⟩ = 1.85), significantly below the bulk value of 2.2. This implies that the first layer of water molecules at a surface are frustrated in their ability to hydrogen bond compared to molecules in the bulk, and this is due to the broken spatial symmetry arising from the presence of the wall. Furthermore, for each ITIM layer in the vicinity of the O-atom wall located at *z* ≈ 5 nm, we calculated the average angle θ′, defined as the angle made between a unit vector representing the average orientation of the water dipole moment and the surface normal **n**_***z***_ (θ′ = cos^–1^(⟨**μ̂**⟩·**n**_*z*_)). Here, values of θ′ above 90° imply an orientational preference of water molecules to point their dipole moments away from (O atoms toward) the surface situated at *z* ≈ 5 nm. Water molecules in the first and second layer make an angle of 92.1° and 90.1° respectively; i.e., there is a slight orientational preference for water molecules in these layers to point their O atoms toward the surface. By the third ITIM layer this value is essentially equal to the bulk water value of 90.0°. Thus, the orientational anistropic effect that gives rise to the excess interfacial electrical potential, φ_int_, is almost exclusively due to the first ITIM layer of water molecules in the immediate vicinity of the surface.

We can further delineate the two types of hydrogen-bonding behavior that are ultimately responsible for the orientational anisostropy at the interface. In particular, we distinguish two distinct hydrogen-bond populations in each ITIM layer that behave as either hydrogen-bond donors or acceptors with other neighboring water molecules. We find that in the first ITIM layer the average orientation of the acceptor water molecules as reflected in their average angle θ′ ≈ 90.5° remains largely unperturbed compared to the bulk, whereas that of the donor molecules is altered more significantly (Figure 2c). Thus, it appears that interfacial water molecules are better able to donate hydrogen bonds when their H atoms are pointing to the bulk rather than toward the interface. The altered orientation of the hydrogen bond donor molecules at an interface is largely responsible for the excess interfacial potential.

### Excess Interfacial Potential and Free Energy at the Silica–Water Interface

In this section we examine the orientation of water molecules at a model hydrophilic silica surface. Compared to a weakly interacting LJ O-atom wall, the orientation of water at a hydrophilic surface is expected to be strongly influenced by the chemical properties of a more realistic surface. In particular, dipole–dipole interactions and hydrogen bonding between water and polar surface groups may be expected to strongly influence the average orientation of water molecules at real surfaces.^68,74^

In our simulations of water at model silica surfaces carrying no net electrical charge we find that profiles in the *z*-component of the dipole moment are more oscillatory with increasing surface silanol group density, as more hydrogen bonds are made with interfacial water molecules (Supporting Information, section 3). Interestingly, despite the additional surface complexity, we note the same overall qualitative behavior for water at an uncharged silica surface as seen for a neutral oxygen atom wall: there is a net orientational anisotropy at the interface, with water molecules slightly preferring to point their oxygen atoms toward the surface. The calculated excess interfacial potential values, φ_0_ ≈ −0.15 to −0.3 V, are therefore also negative in sign and of a similar magnitude to the values obtained for the O-atom wall (Figure 3). Increasing silanol group density on the silica surface supports the ability of silanol groups to hydrogen bond to interfacial water molecules, which in turn introduces competing effects in the average orientation of interfacial water (Figure 3b). The overall result is a smaller net orientational anisotropy at an interface capable of hydrogen bonding, resulting in a smaller magnitude of φ_0_ with increasing silanol group density (Figure 3c). Netz and Janeček have reported similar findings for model surfaces with attached surface hydroxyl groups and further highlight a dependence of φ_0_ on the spatial lateral arrangement of surface groups and the OH bond orientation angle.^76^

We then proceed to calculate the excess interfacial potential as a function of charge density, φ_int_(σ). To modify the surface charge density on the single silica surface in the capacitor setup, we selectively deprotonate surface silanol groups to give a variable net charge density σ; the corresponding φ_int_(σ) values are shown in Figure 3c. Unlike the O-atom wall case, the slope of φ_int_(σ) is more heavily dependent on results obtained for the corresponding continuum electrostatics value, which we discuss in detail in the Supporting Information. We find that as the surface charge increases (corresponding, e.g., to increasing pH in experiment), the net interfacial dipole moment eventually undergoes a reversal in sign, in agreement with the results obtained for the O-atom wall. As the silica surface becomes more negatively charged, a greater number of type 1 hydrogen bonds, *N*_H1_, are formed (in which the oxygen atoms of the surface silanol groups act as hydrogen bond acceptors), whereas the number of type 2 hydrogen bonds, *N*_H2_ (in which the oxygen atoms of the surface silanol groups act as a hydrogen bond donors), decreases (Figure 3b). Water molecules that participate in type 1 hydrogen bonds point their dipole moments toward the surface. In other words, we find that for large negative surface charge densities water molecules point their positive H atoms toward the negative surface, as expected in a simple charge-dipole interaction. This orientational preference generates a more positive interfacial potential.

The calculated excess free energy of solvation, *f*(σ), profiles for our silica systems show good agreement with the original result for an O-atom wall (Figure 3d). In general, we find that silica surfaces with a higher density of silanol groups give nonmonotonic *f*(σ) curves with a lower maximum value of *f*(σ) occurring in the weakly negatively charged regime. The reduction in the value of the maximum and the slight shift to smaller |σ| values points to a mitigation of the net orientational anisotropy of water molecules at strongly hydrogen-bonding surfaces (Figure 3d). The peak in the *f*(σ) curve corresponds to the maximum of the function *f*(σ) = φ_0_σ + *k*σ^2^/2 which is given by σ_max_ = −φ_0_/*k*. We find that all other considerations remaining equal, the value of φ_0_ largely determines that of σ_max_ (see Figure 3c).

Finally, we compare our simulations of the behavior of water an interface to experimental studies. The preferential orientation of interfacial water, where molecules point their O atoms toward the surface, has indeed been observed in nonlinear spectroscopic studies of water structure at weakly charged silica surfaces (corresponding to experiments at low pH).^26,77^ Experimental studies also detect a flip in net orientation of interfacial water molecules as the pH in solution increases (corresponding to an increase in net negative charge density).^26^ Reference (26) proposes a hydrogen-bonding mechanism for the observations which is in good agreement with the simulation results presented in Figure 3b.

### Excess Interfacial Potential and ITIM Analysis at Surfaces of Carboxylated Atactic Polystyrene

We now examine solvation at interfaces of carboxylated atactic polystyrene (PS) (Figure 4a,b). Unlike the systems studied thus far, PS surfaces have considerable roughness resulting in a broadened interfacial region spanning a range in *z* on the order of ≈1 nm. ITIM is useful in the analysis of such systems since it enables the calculation of properties of interest with respect to an instantaneous molecular surface, effectively removing the surface roughness, as shown for one of our model surfaces in Figure 4c. Calculated water density and *P*(*z*) profiles at PS interfaces are in good agreement with a previous study modeling PS melts with attached oxygen atoms.^66^ Note that the surfaces in ref (66) carried neither a net electrical charge nor ionizable groups. In our work we find as in previous studies that water molecules display a slight preferential orientation with their oxygen atoms pointing toward the surface over the large interfacial region. Calculated values of the interfacial potential at zero surface charge, φ_0_, for surfaces with carboxyl group densities of 0.45 and 0.9 nm^–2^ are −0.07 and −0.14 V, respectively. Note that for PS systems we find that the magnitude of φ_0_ increases with increasing density of carboxylic acid surface groups, whereas silica displayed the opposite trend with an increasing density of silanol groups.

**Figure 5:**
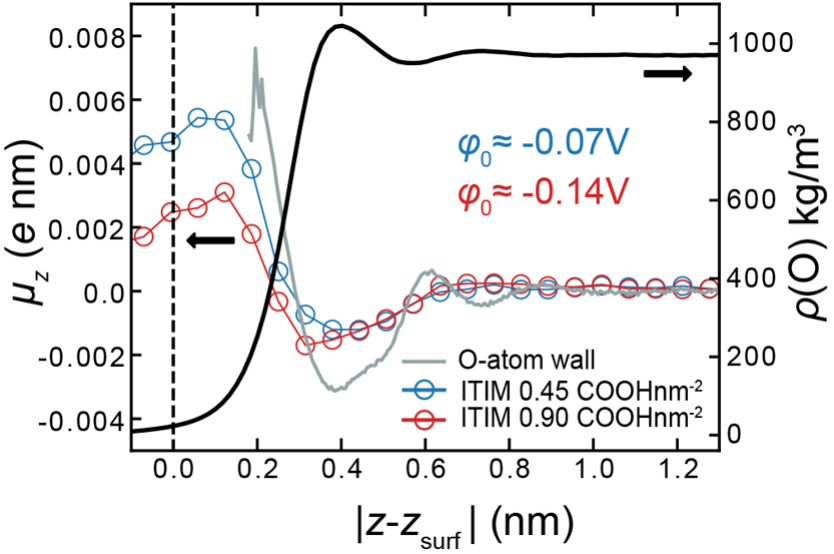
ITIM analysis of water at a carboxylated atactic polystyrene (PS) surface. Dipole moment, μ_*z*_, profiles (left axis), and water density profile (black line, right axis), for water molecules in contact with carboxylated PS calculated as a function of distance from the instantaneous molecular surface generated with the ITIM algorithm. The PS surface topography shown in Figure 4c is now reduced to flat surface, *z*_surf_, in this analysis, represented by the dashed vertical line. Upon removal of the surface roughness, a very similar dipole moment profile emerges as that for water molecules at a simple LJ O-atom wall (gray line). Water molecules in the first hydration layer are again found to be responsible for the orientational anisotropy. Increasing the surface group density results in a progressive change in dipole orientation closest to the surface which ultimately yields an increase in the magnitude of φ_0_ as a function of carboxyl group density. Systems with a higher density of surface groups give values of φ_0_ comparable to water at an O-atom wall.

We provide insight into the origins of the values of φ_0_ by employing the ITIM algorithm.^72^ ITIM analysis for the PS systems reveals that the intrinsic profile for the net *z*-component of the water dipole moment, μ_*z*_(*z*), calculated with respect to the rough PS surface as shown in Figure 4c, is very similar to the μ_*z*_(*z*) profile for water at an O-atom wall (Figure 5). We find that water molecules belonging essentially to the first hydration layer at the interface display a net orientation, pointing their oxygen atoms toward the surface. Subsequent hydration layers do not make a significant contribution to the interfacial potential.

A bare PS surface (devoid of −COOH groups) in general yields very small magnitudes of φ_0_ of about +0.03 V. But experiments involving PS particles always concern polymer material that has some degree of hydrophilic group content, which we have mimicked here by using PS doped with carboxylic acid groups. This system clearly shows negative φ_0_ values, emphasizing the qualitative generality of interfacial water orientation for a range of surface chemistries. Although the orientation of water (as reflected in the dipole moment) at a PS surface reveals the same qualitative orientation as at an O-atom surface, a smaller magnitude of the orientational anisotropy coupled with a reduced peak height in the profile for the density of interfacial water at the PS surface results in an overall diminished value for the interfacial potential φ_0_ (Figure 5).

**Figure 6:**
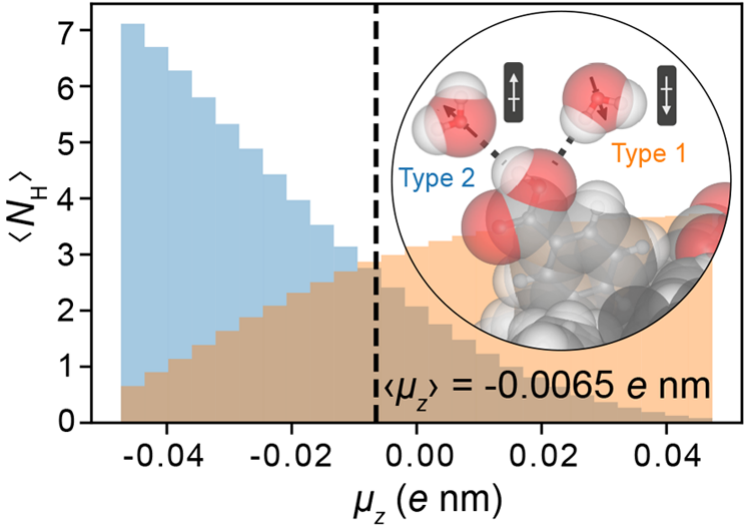
Hydrogen bond analysis of water at a carboxylated PS surface with a carboxyl surface group density of 0.9 OH nm^–2^. Dipole moment distributions of water molecules involved in hydrogen bonds of either type 1 (orange: surface carboxyl group O atoms act as hydrogen bond acceptors) or type 2 (blue: surface carboxyl group O atoms act as hydrogen bond donors). ⟨*N*_H_⟩ denotes the average number of hydrogen bonds per simulation frame. The net *z*-component of the dipole moment of all hydrogen bond orientations is negative, ⟨μ_*z*_⟩ = −0.0065*e* nm (indicated by the black dashed line), i.e., points away from the surface.

We further calculated the orientational distribution of water molecules involved in hydrogen bonds at carboxylated PS surfaces. Given the disordered polymeric surface, carboxyl groups are directed into the solvent at a wide range of angles with respect to the surface normal. Furthermore, because of the large interfacial region, water molecules are now able to solvate surface groups in a different manner to surface groups on our silica surfaces, often spatially surrounding the carboxyl group. Such a surface therefore supports a large distribution of orientations of water molecules involved in hydrogen bonds (Figure 6). The net dipole moment of all water molecules involved in hydrogen bonds is negative, ⟨μ_*z*_⟩ ≈ −0.0065*e* nm (Figure 6, indicated by the black dashed line); i.e., the net dipole moment points away from the surface. This is because type 2 hydrogen bonds (in which the −COOH groups act as H-bond donors, with participating interfacial water molecules on average pointing their oxygen atoms toward the surface) exert a stronger influence on the orientation of water molecules than type 1 hydrogen bonds (that on average support the opposite net orientation) (Figure 6). Silica SiOH groups display the opposite trend with increasing surface group density: silanol groups are found to be more amenable to H-bond acceptor behavior. The overall result of adding carboxyl surface groups to an PS surface is to modulate the dipole moments of interfacial water molecules to point more away from the surface on average (see Figures 5 and 6). This explains the trends in net dipole moments and excess interfacial potential values φ_0_ with increasing surface group density.

### Examination of the Interfacial Potential for Surfaces Immersed in an Aprotic Solvent

Previous studies on asymmetric solvation at ion-sized cavities^25^ have shown that the magnitude and sign of φ_0_ are strongly governed by the “charge-shape asymmetry” of the solvent molecule. Extending this study, we now examine the impact of the solvent on the sign and magnitude of φ_int_ at macroscopic charged surfaces. We consider the solvent DMSO as it is not only aprotic but also displays an “opposite” charge-shape asymmetry to that of water^25^ (i.e., whereas in water the partially positively charged protons are expected to be more exposed than the O atom, in DMSO the electronegative O atom may be considered to be more amenable to interactions with the surrounding solvent than the methyl groups). To study the behavior of DMSO at an interface, we use the OPLS-AA force field^79^ which specializes in the properties of small organic liquids. Not only is the OPLS-AA force field widely used to model DMSO in the literature,^80^ but it is also thermodynamically consistent with the INTERFACE force field, permitting the investigation of inorganic–organic and inorganic–biomolecular interfaces.^62^

We first study the behavior of DMSO in our simple capacitor setup with model O-atom surfaces. We obtain an excess interfacial potential at an uncharged planar surface of φ_0_ ≈ +0.33 V—opposite in sign to the value obtained for water. This is because the dipole moment of interfacial DMSO molecules points toward the surface, which represents the opposite orientation compared to that for water. This value for φ_0_, along with profiles of the free energy per unit area of interfacial solvation, *f*(σ), are found to be fairly consistent across different molecular models for DMSO (Supporting Information, section 6). Furthermore, the sign of the excess interfacial potential, φ_0_, is in agreement with the value previously obtained for an uncharged ion-sized cavity.^25^

**Figure 7:**
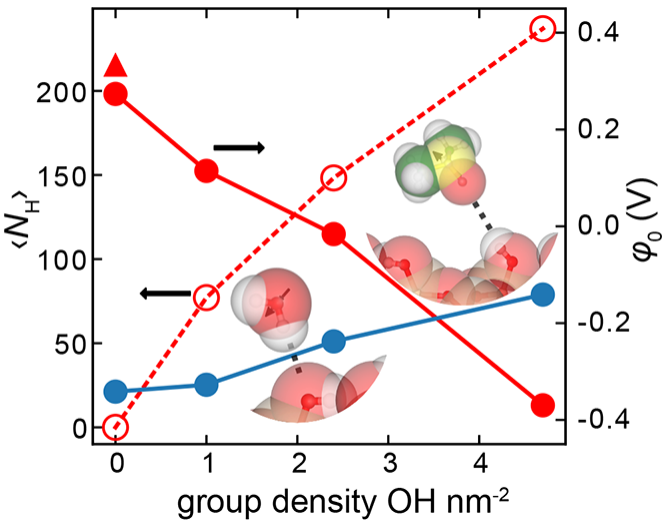
Excess interfacial potential φ_0_ (filled symbols, right axis) for DMSO (red data points) and SPC water (blue data points) in contact with uncharged silica surfaces of varying surface group density. Also shown are the average number of hydrogen bonds per simulation frame for DMSO, ⟨*N*_H_⟩ (open red circles, left axis). The triangle symbol plots the value of φ_0_ for DMSO in contact with an O-atom wall. Also presented is a simulation schematic of a DMSO molecule forming a hydrogen bond with a surface silanol group—the DMSO molecule’s dipole moment points away from the surface. As surface silanol group density increases, ⟨*N*_H_⟩ increases and the value of the potential φ_0_ decreases. Water shows the opposite trend, where φ_0_ increases with increasing surface group density because of the influence of type 2 hydrogen bonds (shown in the water molecule schematic) on interfacial water orientation.

Next we study DMSO in contact with uncharged silica surfaces of varying silanol surface group density. Interestingly, for silica surfaces with a surface silanol group density >2.4 OH nm^–2^, we obtain negative values for excess interfacial potential φ_0_ which is of *opposite* sign to that calculated for DMSO at an O-atom surface and at silica surfaces with lower group densities (Figure 7). Hydrogen-bond analysis reveals that, acting as an acceptor, the −S=O group in DMSO can form strong hydrogen bonds with the −OH surface groups of silica. Such behavior has also been observed in other simulation and nonlinear spectroscopic studies.^81,82^ This hydrogen-bonding picture results in a net orientation of DMSO molecules at silica surfaces of group density >2.4 OH nm^–2^ where the O atoms of DMSO molecules preferentially point toward the silica surface. As we have seen previously, this gives rise to a negative interfacial potential φ_0_. Increasing silica surface group density results in an increase the average number of hydrogen bonds, ⟨*N*_H_⟩, which in turn generates a more negative interfacial potential (Figure 7). Thus, we find that, depending on the solvent, surface chemistry can substantially impact both the sign and magnitude of φ_0_.

## Conclusion

In this study we have examined the influence of surface chemistry on the excess interfacial electrical potential at the solid–liquid interface. We have focused in particular on surfaces capable of hydrogen bonding, e.g., silica and carboxylated PS, as this ability could be expected to substantially impact the average orientation of interfacial solvent molecules. We consistently find the same qualitative results for various types of silica and carboxylated PS surfaces: a majority of interfacial water molecules point their oxygen atoms toward the surface, reflecting the same orientational anisotropy seen at a model O-atom surface. This in turn gives rise to an excess interfacial potential of the same sign but variable magnitude. In our work we find that the sign of this excess potential at a charge free surface (zero ionized surface groups) is negative when referenced with respect to the bulk liquid. Note that this value would imply a positive electric potential when referenced to the wall interior.^22^ The excess potential results in a pronounced nonmonotonic trend in the interfacial free energy with respect to changing surface charge, which has been implicated in the experimentally observed attraction between like charged particles in solution.^19,20^

Overall our work shows that a simple model system consisting of water molecules at an O-atom wall performs well in reproducing the excess free energy of surface solvation of more complex model surfaces.^19^ We have also demonstrated that results for SPC water at an O-atom surface are indeed consistent with a more computationally expensive polarizable water model, AMOEBA14. Careful examination and comparison of hydrogen bonding in layers of interfacial water approaching the surface strongly suggest that the excess interfacial potential indeed arises from the broken hydrogen-bonding symmetry in the presence of an interface. Importantly, we find that the net orientational behavior of water is found to be similar across a range of surface chemistries, which is reflected in a consistently negative value of φ_0_ in the systems we have explored. Nonetheless, we have found that details of surface chemistry can influence the interfacial orientational anisotropy and hence the magnitude of the interfacial potential. For example, for silica with a very high density (4.7 OH nm^–2^) of surface hydroxyl groups, φ_0_ can be smaller by about a factor 2 compared to a situation with a low surface silanol group density (1 OH nm^–2^). We have found however that this trend does not hold in general, and an increase in the number of hydrophilic groups at a hydrophobic PS surface can in fact reinforce the orientational anisotropy, increasing the magnitude of φ_0_, while keeping the same negative sign. These differences in trends arise from structural detail at the interface, the precise nature of the surface groups (carboxyl vs silanol), and the structure of water around the interfacial groups. Despite the rich detail in the underlying phenomenology, the final result as embodied in the magnitude and sign of φ_0_ for water at an interface seems to consistently point to a value of φ_0_ ranging between −0.1 and −0.3 V. Finally, the value of φ_0_ of course depends on the solvent species in question. For an aprotic solvent such as DMSO we have found that φ_0_ can even change sign depending on the hydrogen-bonding ability of the surface. In general, isotropic symmetry in bonding interactions between solvent molecules, which is broken at an interface, will result in nonzero values of the interfacial potential as demonstrated in previous studies.^21,25,47^

Interfacial water has long been known to play a major role in short-range hydration forces^83−86^ and more broadly in the thermodynamics of molecular binding interactions.^12,13^ We provide a framework within which the excess interfacial potential, φ_int_(σ), and therefore the excess solvation free energy per unit area, *f*(σ), has a significant impact on interparticle and intermolecular interactions in solution. The surface charge density of an ionizable surface in solution can respond to the approach of a charged object at very large distances due to a phenomenon called charge regulation.^41,42,84^ Owing to the response of interfacial–water orientation to the interfacial charge density, we expect a solvation contribution to the total interparticle interaction energy or force. Because of the intrinsic coupling of the surface charge density to the electrostatic interaction, the interfacial solvation free energy contribution is expected to be as long-ranged as the electrostatic interaction and governed by effectively the same decay length, κ^–1^, which can be on the order of hundreds of nanometers in solutions of low ionic strength.^20^ Such a contribution is fundamentally different from short-range hydration forces which arise from a more direct steric interaction mechanism.^83,85^ The molecular level findings reported in this work underscore the generality, and the qualitative insensitivity to surface chemistry, of the long-range interparticle attraction observed for negatively charged matter dispersed in water.^19,34^

Although we have discussed the problem in the context of interparticle interactions, the remit of such behavior is not limited to macroscopic interfaces. Indeed, we expect the same considerations to hold in the interactions of biomolecules and biological interfaces in solution. The proposed interfacial mechanism is likely to find relevance in a broad range of phenomena such as biological phase segregation,^87,88^ crystallization and pH-induced gelation, and chromosome packing or more generally in soft-matter and molecular biological systems that exhibit pH and salt concentration dependent attractive interactions between entities carrying negative electrical charge. Such behavior is indicative of a tunable, attractive interaction that may well find its roots in the orientational behavior of water at the molecular interface in solution.

## Supporting Information Available

Details on the calculation of the interfacial potential, extended analysis, and further characterization of the molecular models employed (PDF)
