# Supplementary material for: The Role of Surface Chemistry in the Orientational Behavior of Water at an Interface
Source: J Phys Chem B. 2022 Jun 21;126(25):4697–710. doi: 10.1021/acs.jpcb.2c01752 (PMC9251758; doi:10.1021/acs.jpcb.2c01752)
Supplement: Supplementary file 1 — jp2c01752_si_001.pdf [file jp2c01752_si_001.pdf]

The Role of Surface Chemistry in the Orientational Behaviour of Water at an Interface

Rowan Walker-Gibbons,[†] Alžbeta Kubincová,[‡] Philippe H. Hünenberger,[‡] and Madhavi Krishnan[*,†]

†*Physical & Theoretical Chemistry Laboratory, Department of Chemistry, South Parks Road, University of Oxford, Oxford OX1 3QZ, United Kingdom*

‡*Laboratory of Physical Chemistry, Department of Chemistry and Applied Biosciences, ETH Zurich, Vladimir-Prelog-Weg 2, CH-8093 Zürich, Switzerland*

E-mail: madhavi.krishnan@chem.ox.ac.uk

Supporting Information Available

1. Calculation of the Interfacial Electric Potential from Classical MD Simulations

The z-component of the dipole moment vector, $\mu_z(z)$, of SPC water molecules in the capacitor setup can be written as:

$$\mu_z(z) = \langle \sum_{i=\text{molecules}} \sum_{j=1} q_j (z - z_{O,j}) \rangle = |\mu| \langle \cos\theta_z \rangle \tag{1}$$

where the index i runs over all water molecules and the index j over each atom in the water molecules. The angular brackets denote an ensemble average and $z_{O,j}$ is the z-coordinate of the oxygen atom of the corresponding water molecule. The magnitude of the dipole moment $|\mu|$ for the SPC water model is 2.27 Debye. $\langle \cos(\theta_z) \rangle$ denotes the average cosine of the angle between the water dipole moment vector the surface normal, $\boldsymbol{n_z}$, that points away from the oxygen atom wall (located on the left side of the capacitor) in the positive z-direction, as shown in Figure 1a. The total polarization $P(z)$ contains contributions from both the dipole moment density, $P_1(z) = \rho(z)\mu_z(z)$ (where $\rho(z)$ is the average local density of water molecules), and a traceless quadrupole moment density Q_{zz}.[1] The electrical potential at z is then given by:

$$\varphi(z) = \frac{1}{\epsilon_0} \int_{z_{\text{mid}}}^{z} \left(P_1(z) - \frac{\mathrm{d}Q_{zz}(z)}{\mathrm{d}z'} \right) \mathrm{d}z \tag{2}$$

where

$$Q_{zz}(z) = \frac{\rho(z)}{2} \left\langle \sum_{i=\text{ molecules}} \sum_{j=1} q_j \left[(z - z_{O,j})^2 - \frac{1}{3} (\mathbf{r} - \mathbf{r}_{O,j})^2) \right] \right\rangle \tag{3}$$

is the traceless quadrupole moment density, with $\mathbf{r} = \sqrt{x^2 + y^2 + z^2}$.[1,2] In practice, we only calculate the potential arising from the dipole moment density $P_1(z)$. The traceless quadrupole moment density Q_{zz} is zero both in the bulk liquid, where solvent molecules are randomly oriented, and tends to zero at the interface with the solid phase, where the density of solvent molecules vanishes. Therefore evaluation of the contribution to the interfacial potential φ_{int} due to the traceless quadrupole density via eq 1, is approximately zero $(Q_{zz}|_{z_s} - Q_{zz}|_{z_{mid}} \approx 0)$ (Figure S1).

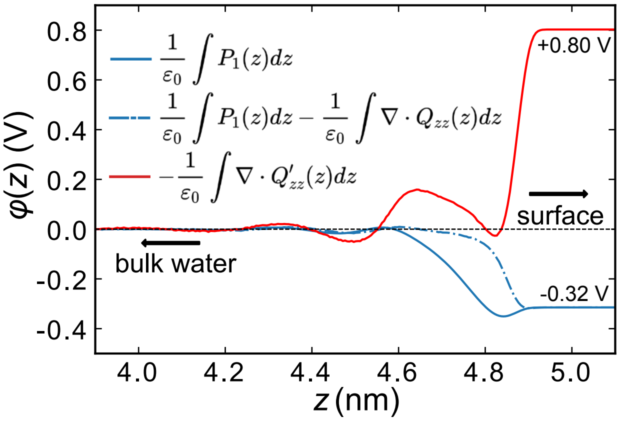

Figure S1: Components of of the electric potential across an uncharged model O-atom surface in contact with water. The dipole moment density $P_1(z)$ (solid blue line) gives an interfacial potential φ_{int} of -0.32 V at the surface. Although the *traceless* quadrupole density, $Q_{zz}(z)$, is non-zero close to the interface, inclusion of this contribution does not change this final value of φ_{int} (dashed blue line, calculated using eq 1). It is worth noting that the electrical potential due to *non-traceless* quadrupole moment density Q'_{zz} entails a value of +0.8 V at the surface (red line). All potential values are calculated with respect to a reference location in the bulk liquid.

Inclusion of the contribution to the electrical potential due to the quadrupole moment density $Q'_{zz}(z)$ of the SPC water molecules can give rise to a potential that is large and of opposite sign compared to that generated by the dipole moment density $P_1(z)$. However, as we discuss in the main text, this term should not be considered when calculating the electrical potential from classical MD simulations. Only the traceless quadrupole moment density $Q_{zz}(z)$ contributes to the potential felt by a test charge located external to the solvent molecule.[1]

Dependence of the Dipole Contribution to the Interfacial Potential on the Choice of Molecular Center

Determination of the interfacial potential φ_{int} using eq 1, via integration of the dipole polarization $P_1(z)$, has been shown previously to be dependent on the choice of a molecular center used to locate molecules in the analysis - a known issue of employing an M-type cutoff.[3–7] This is simply because a dividing surface, imposed at z_{mid} in our case, sorts molecules based on the location of their center to lie inside or outside of the region bounded by the surface and z_{mid}. Slightly different populations of molecules will result for each center, which in turn introduces a bias in the calculation of the interfacial potential which can be of considerable magnitude, especially for water.[6,7]

Rather than specifying an arbitrary molecular center by which to locate molecules, the ITIM algorithm can serve as a more robust method to select a population of interfacial molecules. ITIM selects a molecule based on whether a van der Waals sphere of an atom (the radii of which are taken from the forcefield used to perform the simulation) in that molecule has been hit by a probe sphere travelling perpendicular to the surface of interest,[8] as described in the main text. Calculation of the dipole contribution to the interfacial potential on a consistent group of molecules generated with the ITIM algorithm will give the same result, irrespective of the molecular center used in the analysis.

We note that the calculation of φ_{int} on a group of molecules generated with the ITIM algorithm is different to the approach taken in previous work where a molecular center was chosen for solvent models containing more than one vdW site.[9] In the current work, we perform this novel analysis only on simulations involving DMSO at our silica surfaces. We determine the number of molecular ITIM layers to be generated in the analysis such that the contribution of the final ITIM layer alone φ_{int} is approximately zero. We point out that the calculated value of φ_{int} for SPC water ($\varphi_0 \approx -0.3$ V) is independent of the method used, since SPC water has only one vdW site (the oxygen atom).

2. Implementation of the AMOEBA14 Water Model

The Dipole Moment for AMOEBA Water

The dipole moment for a water molecule described by the AMOEBA polarizable model has three contributions: the dipole generated by the atomic partial charges ($\Sigma q_j(z - z_{O,j})$), the permanent atomic dipoles and the induced atomic dipoles. The permanent and induced dipoles are located on every atomic site, giving a total of 7 vectors contributing to the total dipole moment for any given water molecule (Figure S2). In the main text, we binned the dipole generated by the atomic partial charges on the oxygen atom coordinate of the water molecules (as was done for SPC water), and binned the vector sum of the permanent and induced dipoles, which are located on every atomic site, on the center of geometry of the water molecules. Generation of the polarization $P(z)$ in this way results in an excess potential profile $\varphi_{\text{int}}(\sigma)$ that is very close to the result of SPC water (Figure S2). However, we note that the $\varphi_{\text{int}}(\sigma)$ profile can change substantially if $P(z)$ is instead calculated by binning the vector sum of all seven contributions to the molecular dipole moment at the location of each water molecule's oxygen atom or alternatively, on the center of geometry of the molecule (Figure S2, red and orange data points respectively). Despite this, we point out that the overall qualitative behaviour remains consistent.

Treatment of Long-Range Electrostatics: The 3dc Correction

Performing a conventional Ewald summation to calculate the long-range electrostatics for interfacial systems that adopt slab geometry leads to the artificial polarization of molecules in the simulation box due to neighbouring periodic images.[10–12] To address this, we left a vacuum gap of twice the slab length in the z-direction of our simulation cells and implemented the 3dc of correction of Yeh & Berkowitz.[10] This amounts to applying an external force to each atomic site of the form:

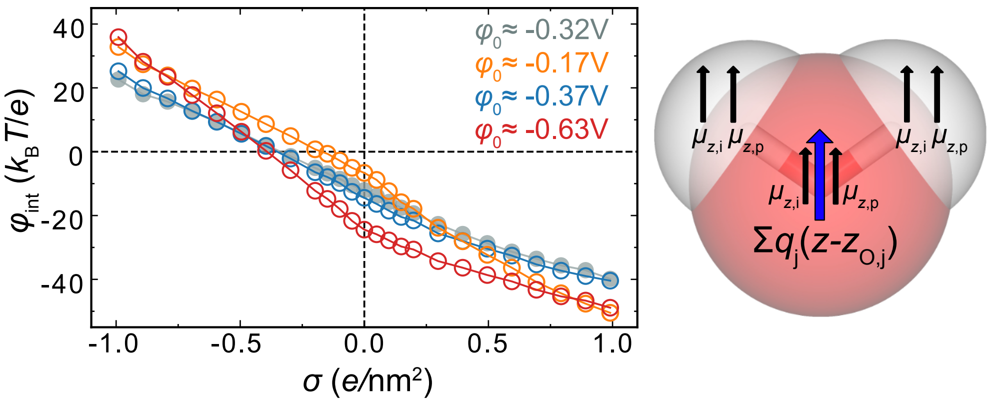

Figure S2: Influence of the binning scheme of the molecular dipole moments on value of the excess interfacial potential φ_{int} for water described by the AMOEBA14 model at an uncharged O-atom surface. The dipole generated by the atomic partial charges $(\Sigma q_j(z - z_{O,j}))$ is binned at the location of the oxygen atom of each water molecule and the vector sum of all six permanent $(\mu_{z,p})$ and induced dipole vectors $(\mu_{z,i})$ on the water molecule's center of geometry (blue data points). The vector sum of all contributions to the molecular dipole moment are binned at the oxygen atom coordinate and center of geometry of the water molecules respectively (red and orange data points). $\varphi_{\text{int}}(\sigma)$ obtained for SPC water is plotted for comparison (grey data). Values for the excess interfacial potential at zero surface charge, φ_0, are quoted for each case.

$$\boldsymbol{F}_j = \frac{4\pi M_z}{V} q_j \hat{\boldsymbol{z}} \tag{4}$$

where $M_z = \sum_j q_j z_j$ is the z-component of the total system dipole moment and V is the volume of the simulation cell. For simulations involving AMOEBA water, the true system dipole also includes contributions from the permanent and induced dipole moments on each atomic site of the molecules. However, we find that they do not make a large contribution to the total system dipole moment, M_z, as a whole. We find that the approximate correction of eq 4 is able to yield values for the permittivity in the middle of the capacitor ϵ_{mid} that are very close to the expected value for a capacitor with continuum water as the intervening dielectric. However, we find that the ϵ_{mid} values are consistently larger than the bulk value of 80 by a few percent.

3. Further Characterisation of the Silica-Water Interface

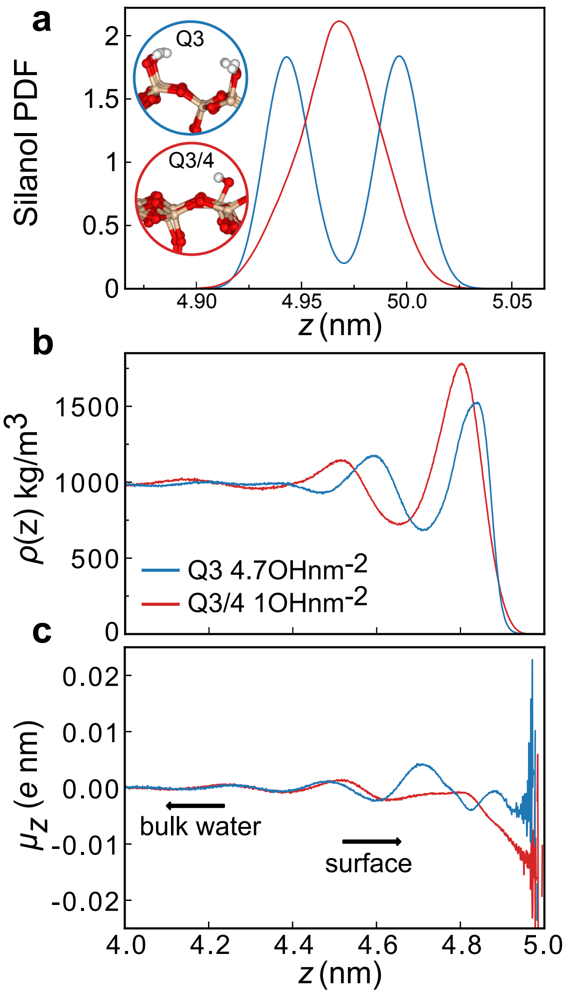

Figure S3: (a) Spatial probability density distributions (PDFs) for silanol groups at silica surfaces. Unlike a flat O-atom wall, the silanol groups on our silica surfaces display some spatial distribution in z. We note a bimodal distribution of surface silanols for a Q3 surface carrying 4.7OH nm^{-2} due to the vicinal silanols and which disappears with decreasing silanol group density. (b) Average water density, ρ(z), profiles at silica surfaces of varying surface group density. (c) Corresponding water dipole moment profiles for the two silica surface types. A more oscillatory dipole moment profile is found for water molecules at hydrophilic silica surfaces (4.7OH nm^{-2}), where there is increased hydrogen bonding between interfacial water and polar surface groups.

4. Construction of a Continuum Electrostatics Model for the Silica-Water Interface

An important aspect of the approach to determine the excess interfacial potential, particularly in systems containing more realistic descriptions of surfaces, concerns the form of the continuum model dielectric function. In simulations where the walls carry a non-zero surface charge density, the polarisation from the continuum electrostatics model is subtracted from the MD polarisation profile in order to obtain the net 'excess' interfacial potential, φ_{int}, as discussed in the main text. The definition of this function can therefore have a substantial impact on the final integrated value of φ_{int}.

Unlike the oxygen atom wall, our silica surfaces do not present sharp discontinuities at an interface with a solvent, with the silanol groups and siloxane bridges displaying some degree of spatial distribution in z of $\approx$1 Å width (Figure S3). This gives rise to an interfacial region that contains both surface atoms and interfacial solvent molecules. The local value of the permittivity is also expected to be smaller in this interfacial region.[13] This in turn implies a smaller net polarisation $P(z)$ compared to the bulk. In our work we determine an interfacial plane that delimits the bulk region where $\epsilon = \epsilon_{\text{mid}}$ from the interfacial zone where we set $\epsilon = 1$ (Figure S4), as described in the main text. This approach works well for silica where the surface charge may be considered to lie on a well defined plane in the parallel-plate set-up, however it might not applicable to a substantially more disordered surface such as PS which displays a large interfacial region populated with charges distributed over a wide range of distances from the nominal Gibbs dividing surface.

For water at our model silica surfaces, a change in the location of the interfacial plane z_{int} (which can also be thought of as a change in the profile of the permittivity ϵ at the interface) can change the obtained value of the interfacial potential φ_{int} significantly (Figure S4). However, this ambiguity arises only in systems with net charge-carrying walls and does not affect the value of φ_0 obtained for walls with $\sigma = 0$, where the absence of charge implies that there is no continuum contribution to the electrostatics to consider. In the

regime of low to moderate surface charge densities of interest in most experimental situations ($\sigma \lesssim 0.1 e\text{nm}^{-2}$), we have shown that the excess hydration free energy can be approximated as $f(\sigma) \approx \varphi_0 \sigma$. As a result we can assume that the ambiguity in a continuum model of the interfacial dielectric function and the choice of z_{int} does not affect comparisons of simulation interfacial free energies with experimental data at low surface charges densities typical for experiments.

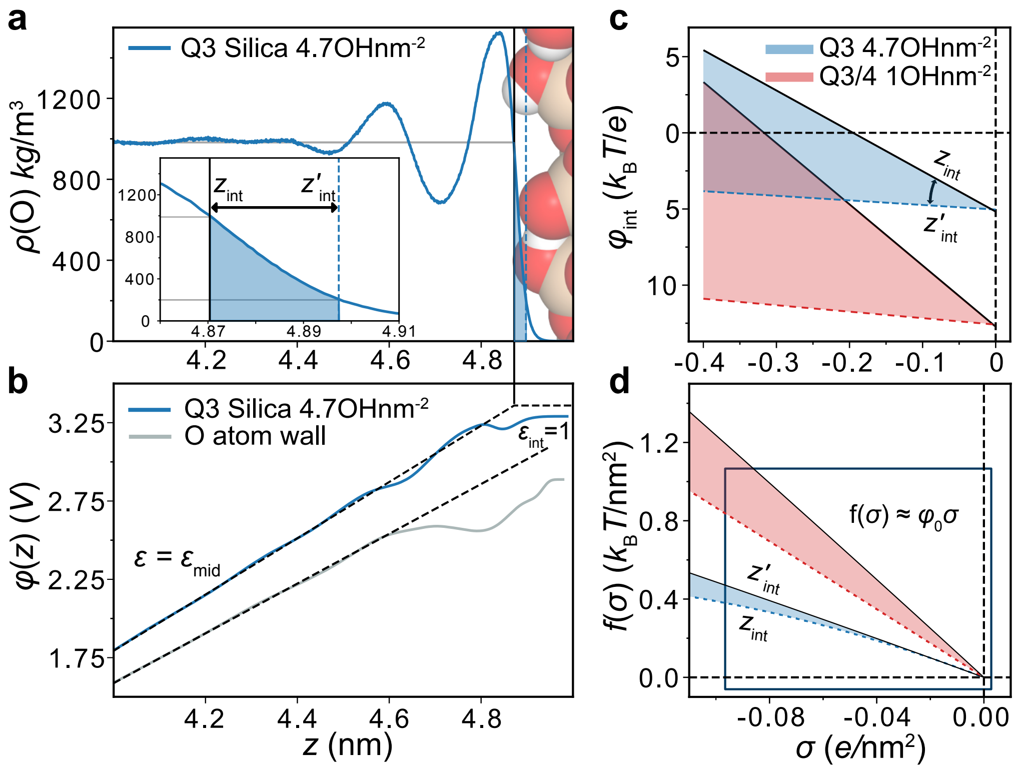

Figure S4: Location of the interfacial plane z_{int} and its influence on the calculation of the excess interfacial potential φ_{int} for charged silica surfaces. (a) Water density profile, $\rho(\text{O})$, at a silica surface with silanol surface group density 4.7 OH nm^{-2}. The interfacial plane, z_{int}, (solid black vertical line) is defined to be at the location where the water density finally falls below the bulk value. This location coincides with the onset of surface silanol group hydrogen atoms. The location of z_{int} is somewhat arbitrary and could similarly be defined to be at z'_{int} (dashed blue vertical line). (b) The potential profile $\varphi(z)$ from the bulk liquid up to a charged silica surface (blue line) and O-atom wall (grey line) in a capacitor with walls that have a charge density of $\sigma \approx \pm 0.1 e\text{nm}^{-2}$. Black dashed lines show the estimated electrical potential profile from the continuum electrostatics models for each case where we assume a bulk value for the relative dielectric constant, ϵ_{mid}, up to an interfacial plane z_{int}. Beyond z_{int} for the silica surface, $\epsilon = \epsilon_{\text{int}} = 1$ in the interfacial zone (flat region of the line). (c) The range of possible excess interfacial potentials φ_{int} for two silica surfaces of different surface group density for two limiting values of the location of the interfacial plane given by z_{int} and z'_{int}, as shown inset in (a)). (d) The corresponding profiles for the free energy of surface solvation $f(\sigma)$ given the range of possible locations of the interfacial plane. In the limit of very low charge densities, $f(\sigma) \approx \varphi_0 \sigma$, rendering the magnitude of $f(\sigma)$ less dependent on the exact location of the interfacial plane.

5. Modelling Carboxylated PS Systems

a

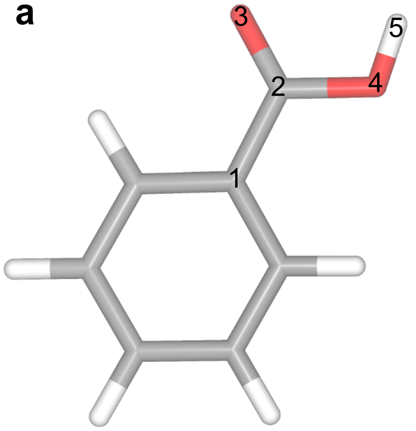

b

No.	CGenFF atom type	Partial charge *e*
1	CG2R61	0.080
2	CG202	0.460
3	OG2D1	-0.460
4	OG311	-0.510
5	HGP1	0.430

Total = 0 *e*

Figure S5: Modification of surface styrene rings in order to attach carboxyl groups (a) To add carboxyl groups to the styrene rings, a hydrogen bonded in the ortho or meta positions on the ring is first randomly chosen and removed. Then the carboxyl group atoms are added in the plane of the ring. Finally, the bond, angle, charge, pair and dihedral information is updated and written to the topology. (b) Atom type and partial charge information for the added/modified atoms.

Quantification of the surface roughness of model PS surfaces

Similar to previous work,[14] we characterised the surface roughness of our carboxylated PS surfaces by calculating the root mean square roughness $R_q = \sqrt{1/N \sum_{i=1}^{N} \Delta z_i^2}$. Here, Δz is the distance of each of the N surface atoms from the Gibbs dividing surface (GDS), which is the thermodynamically defined interface position, defined as:

$$z^{\text{GDS}} = z_{\text{s}} - \int_{z_{\text{s}}}^{z_{\text{l}}} \frac{\rho(z_{\text{l}}) - \rho(z)}{\rho(z_{\text{l}}) - \rho(z_{\text{s}})} \tag{5}$$

where $\rho(z_{s/l})$ are values of the density of PS at positions deep in the solid ($\approx$ 1kgm^{-3}) and liquid phases (0 kgm^{-3}) respectively. We calculate $R_q \approx 0.37$nm in water for both surfaces with carboxyl group densities 0.45 and 0.90 OHnm^{-2}. This value is in close agreement with a previous simulation study and experimental AFM scans of spincoated PS surfaces.[14]

6. Excess Interfacial Solvation Free Energy for DMSO in the O-atom capacitor Setup

Here we examine the excess interfacial solvation free energy for the OPLS-AA DMSO model in our O-atom capacitor setup. We calculated the excess interfacial potential at zero surface charge to be $\varphi_0 = +0.33$ V using the ITIM method (described in Supporting Information Section 1) or $\varphi_0 = +0.45$ V if the center of geometry of the DMSO molecules is used as the molecular center. This value is opposite in sign but of similar magnitude to that of water ($\varphi_0 \approx -0.3$ V). We further find that the net orientational response of interfacial DMSO molecules to increasing surface charge is stronger than for water. This is reflected in the value of $\sigma_{\mathrm{max}} = \varphi_0/k \approx 0.15e$ nm^{-2} which is around a factor 2 smaller for DMSO than water (Figure S6). We also studied the 2016h66 united atom model of DMSO[15] in our O-atom capacitor setup and obtained $\varphi_0 = +0.25$ V which is similar to the value from the OPLS-AA model. Overall we found that the $\varphi_{\mathrm{int}}(\sigma)$ profile and hence the excess free energy of interfacial solvation for DMSO was relatively insensitive to the molecular model studied.

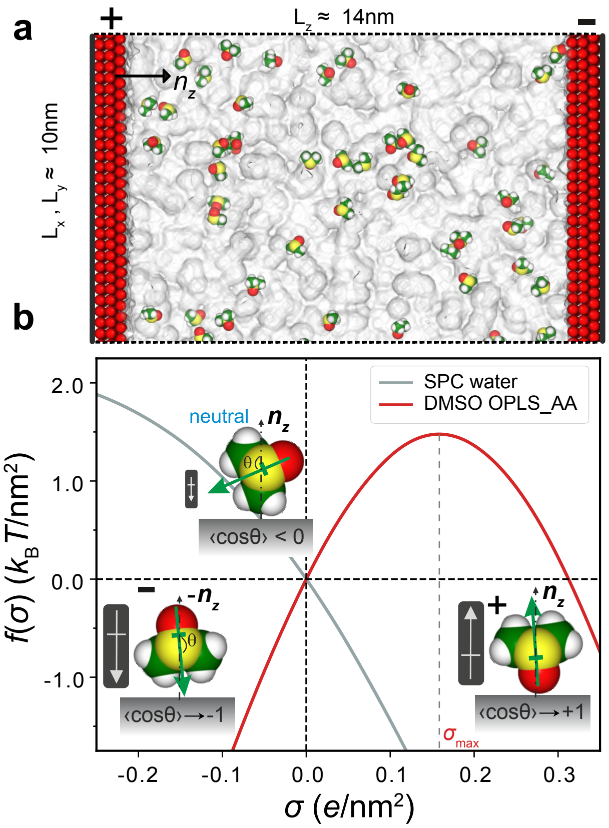

Figure S6: Excess hydration free energy of a charged interface in contact with OPLS-AA DMSO. (a) Schematic representation of the capacitor simulation cell, with 10014 DMSO molecules confined between the two plates separated by a distance of 12 nm. (b) The excess hydration free energy per unit area for DMSO (red curve) compared with the SPC water result (grey curve). The molecular orientation schematics show that at a neutral interface, DMSO molecules preferentially point their dipole moments towards the surface, which is opposite to the orientation observed for water, and which gives a positive value of φ_0. The resulting non-monotonic behaviour for $f(\sigma)$ is expected to be in the weakly *positively* charged regime for DMSO as opposed to the negatively charged regime for SPC water.

References

(1) Cendagorta, J. R.; Ichiye, T. The Surface Potential of the Water–Vapor Interface from Classical Simulations. *The Journal of Physical Chemistry B* **2015**, *119*, 9114–9122.

(2) Stone, A. J. *The Theory of Intermolecular Forces*; Clarendon: Oxford, U.K., 1996.

(3) Hummer, G.; Pratt, L. R.; García, A. E.; Berne, B. J.; Rick, S. W. Electrostatic Potentials and Free Energies of Solvation of Polar and Charged Molecules. *The Journal of Physical Chemistry B* **1997**, *101*, 3017–3020.

(4) Hummer, G.; Pratt, L. R.; García, A. E.; Garde, S.; Berne, B. J.; Rick, S. W. Reply to Comment on "Electrostatic Potentials and Free Energies of Solvation of Polar and Charged Molecules". *The Journal of Physical Chemistry B* **1998**, *102*, 3841–3843.

(5) Kastenholz, M. A.; Hunenberger, P. H. Computation of methodology-independent ionic solvation free energies from molecular simulations. I. The electrostatic potential in molecular liquids. *J Chem Phys* **2006**, *124*, 124106.

(6) Duignan, T. T.; Baer, M. D.; Schenter, G. K.; Mundy, C. J. Electrostatic solvation free energies of charged hard spheres using molecular dynamics with density functional theory interactions. *The Journal of Chemical Physics* **2017**, *147*, 161716.

(7) Cox, S. J.; Thorpe, D. G.; Shaffer, P. R.; Geissler, P. L. Assessing long-range contributions to the charge asymmetry of ion adsorption at the air–water interface. *Chemical Science* **2020**, *11*, 11791–11800.

(8) Pártay, L. B.; Hantal, G.; Jedlovszky, P.; Vincze, A.; Horvai, G. A new method for determining the interfacial molecules and characterizing the surface roughness in computer simulations. Application to the liquid–vapor interface of water. *Journal of Computational Chemistry* **2008**, *29*, 945–956.

(9) Reif, M. M.; Hünenberger, P. H. Origin of Asymmetric Solvation Effects for Ions in Water and Organic Solvents Investigated Using Molecular Dynamics Simulations: The Swain Acity-Basity Scale Revisited. *Journal of Physical Chemistry B* **2016**, *120*, 8485–8517.

(10) Yeh, I. C.; Berkowitz, M. L. Ewald summation for systems with slab geometry. *Journal of Chemical Physics* **1999**, *111*, 3155–3162.

(11) Yeh, I.-C.; Wallqvist, A. Cite as. *The Journal of Chemical Physics* **2011**, *134*, 55109.

(12) Bostick, D.; Berkowitz, M. L. The Implementation of Slab Geometry for Membrane-Channel Molecular Dynamics Simulations. *Biophysical Journal* **2003**, *85*, 97–107.

(13) Bonthuis, D. J.; Gekle, S.; Netz, R. R. Profile of the static permittivity tensor of water at interfaces: consequences for capacitance, hydration interaction and ion adsorption. *Langmuir* **2012**, *28*, 7679–94.

(14) Andrea Muntean, S.; Kemper, M.; van IJzendoorn, L. J.; Lyulin, A. V. Roughness and Ordering at the Interface of Oxidized Polystyrene and Water. **2011**, *27*, 8678–8686.

(15) Horta, B. A. C.; Merz, P. T.; Fuchs, P. F. J.; Dolenc, J.; Riniker, S.; Hünenberger, P. H. A GROMOS-Compatible Force Field for Small Organic Molecules in the Condensed Phase: The 2016H66 Parameter Set. *Journal of Chemical Theory and Computation* **2016**, *12*, 3825–3850.
